# Honeybee venom and melittin suppress growth factor receptor activation in HER2-enriched and triple-negative breast cancer

**DOI:** 10.1038/s41698-020-00129-0

**Published:** 2020-09-01

**Authors:** Ciara Duffy, Anabel Sorolla, Edina Wang, Emily Golden, Eleanor Woodward, Kathleen Davern, Diwei Ho, Elizabeth Johnstone, Kevin Pfleger, Andrew Redfern, K. Swaminathan Iyer, Boris Baer, Pilar Blancafort

**Affiliations:** 1 School of Human Sciences, The University of Western Australia, Perth, WA 6009 Australia; 2 Cancer Epigenetics Group, Harry Perkins Institute of Medical Research, Perth, WA 6009 Australia; 3 Plant Energy Biology, The University of Western Australia, Perth, WA 6009 Australia; 4 Centre for Medical Research, The University of Western Australia, Perth, WA 6009 Australia; 5 Monoclonal Antibody (MAb) Facility, Harry Perkins Institute of Medical Research, Perth, WA 6009 Australia; 6 School of Molecular Sciences, The University of Western Australia, Perth, WA 6009 Australia; 7 Molecular Endocrinology and Pharmacology, Harry Perkins Institute of Medical Research, Perth, WA 6009 Australia; 8 Australian Research Council Centre for Personalised Therapeutics Technologies, Perth, Australia; 9 Dimerix Limited; Nedlands, Perth, WA 6009 Australia; 10 School of Medicine, The University of Western Australia, Perth, WA 6009 Australia; 11 Centre for Integrative Bee Research (CIBER), Department of Entomology; University of California Riverside, Riverside, CA 92521 USA; 12 The Greehey Children’s Cancer Research Institute, The University of Texas Health Science Center at San Antonio, San Antonio, TX 78229 USA

**Keywords:** Breast cancer, Breast cancer, Molecular medicine

## Abstract

Despite decades of study, the molecular mechanisms and selectivity of the biomolecular components of honeybee (*Apis mellifera*) venom as anticancer agents remain largely unknown. Here, we demonstrate that honeybee venom and its major component melittin potently induce cell death, particularly in the aggressive triple-negative and HER2-enriched breast cancer subtypes. Honeybee venom and melittin suppress the activation of EGFR and HER2 by interfering with the phosphorylation of these receptors in the plasma membrane of breast carcinoma cells. Mutational studies reveal that a positively charged C-terminal melittin sequence mediates plasma membrane interaction and anticancer activity. Engineering of an RGD motif further enhances targeting of melittin to malignant cells with minimal toxicity to normal cells. Lastly, administration of melittin enhances the effect of docetaxel in suppressing breast tumor growth in an allograft model. Our work unveils a molecular mechanism underpinning the anticancer selectivity of melittin, and outlines treatment strategies to target aggressive breast cancers.

## Introduction

The European honeybee (*Apis mellifera*) has been the source of a number of products used medicinally by humans, such as honey, propolis, and venom for thousands of years^1^. However, the molecular determinants of the anticancer activity of bee venom remain poorly understood, particularly in breast cancer, the most common cancer in women worldwide^2^. Understanding the molecular basis and specificity of bee venom against cancer cells is key for developing and optimizing novel effective therapeutics from a natural product that is widely available and cost-effective to produce in many communities around the world.

The active component of honeybee venom is melittin, comprising half of honeybee venom by dry weight^3,4^. Melittin is a positively charged, amphipathic 26-amino-acid peptide^5^ that associates with the phospholipids of the membrane bilayer, causing cell death by forming ~4.4 nm-diameter transmembrane toroidal pores that may enable the internalization of additional small molecules with cytotoxic activities^4,6,7^.

Both honeybee venom and melittin have demonstrated antitumoral effects in melanoma^8^, non-small-cell lung cancer^9^, glioblastoma^10^, leukemia^11^, ovarian^12^, cervical^13^, and pancreatic cancers^14^, with higher cytotoxic potency in cancer cells compared to nontransformed cells^8,11,14,15^. Melittin nanoparticles have been used to suppress liver metastasis through the immunomodulation of liver sinusoidal endothelial cells^16^. Additive and synergistic anticancer effects have been reported between honeybee venom and other therapeutic modalities, including with cisplatin in cervical and laryngeal malignancies^17^, and with docetaxel in lung cancer cells^18^. Similar interactions have been demonstrated between melittin and plasma-treated phosphate-buffered saline in MCF7 breast cancer and melanoma cells^19^. Honeybee venom and melittin also induced apoptosis in MCF7 cells^20^, and reduced cell viability and migration in MDA-MB-231 breast cancer cells^21,22^. Honeybee venom reduced metastases of breast cancer to the lung^23^, inhibited tumor growth, and prolonged survival in mice with spontaneous mammary carcinoma tumors^24^. The majority of the antineoplastic activity of honeybee venom has been attributed to melittin^25^ through inhibition of the PI3K/Akt/mTOR axis in breast cancer^21^, MAPK in melanoma^26^, JAK2/STAT3 in ovarian cancer^12^, and NFκB signaling pathways in lung carcinoma cells^18^. In contrast to honeybee venom, bumblebee (*Bombus terrestris*) venom does not contain melittin^27^, but contains secretory phospholipase A2 that induced apoptosis by inhibition of Akt phosphorylation in human chronic myelogenous leukemia cells^28^.

To the best of our knowledge, the effects of different bee venoms and melittin across breast cancer subtypes compared to nontransformed cells have not been investigated. Triple-negative breast cancers (TNBCs, lacking the expression of estrogen and progesterone receptors, as well as human epidermal growth factor receptor 2, HER2^29^) are aggressive and associated with the poorest outcomes^30–33^. Approximately 50% of TNBCs overexpress epidermal growth factor receptor (EGFR)^34^, and HER2-enriched tumors overexpress HER2, another receptor tyrosine kinase (RTK) that confers oncogenic signaling often dependent on the PI3K/Akt pathway downstream^34^. Blocking EGFR signaling in TNBC with standard therapies has demonstrated limited clinical efficacy in early-phase clinical trials due to a lack of dependence on the EGFR pathway and the importance of collateral pathways^35^. Although HER2-targeted therapies have dramatically improved median survival in the metastatic setting, resistance is also almost inevitable over the longer term for this subtype^33,36^. Clearly, the discovery of more effective and selective therapeutic strategies for these cancers is a priority area in clinical oncology.

Here, we report that honeybee venom and melittin induce potent and highly selective cell death in TNBC and HER2-enriched breast carcinoma with negligible effects in normal cells, by interfering with growth factor-dependent RTK interactions critical for receptor phosphorylation and activation of PI3K/Akt signaling. Beyond breast cancer, we also outline targeted modifications of melittin for potential use in combination with chemotherapy for the treatment of other aggressive cancers driven by overexpression of growth factor receptors.

## Results

### Honeybee venom and melittin reduce breast cancer viability

**Fig. 1:**
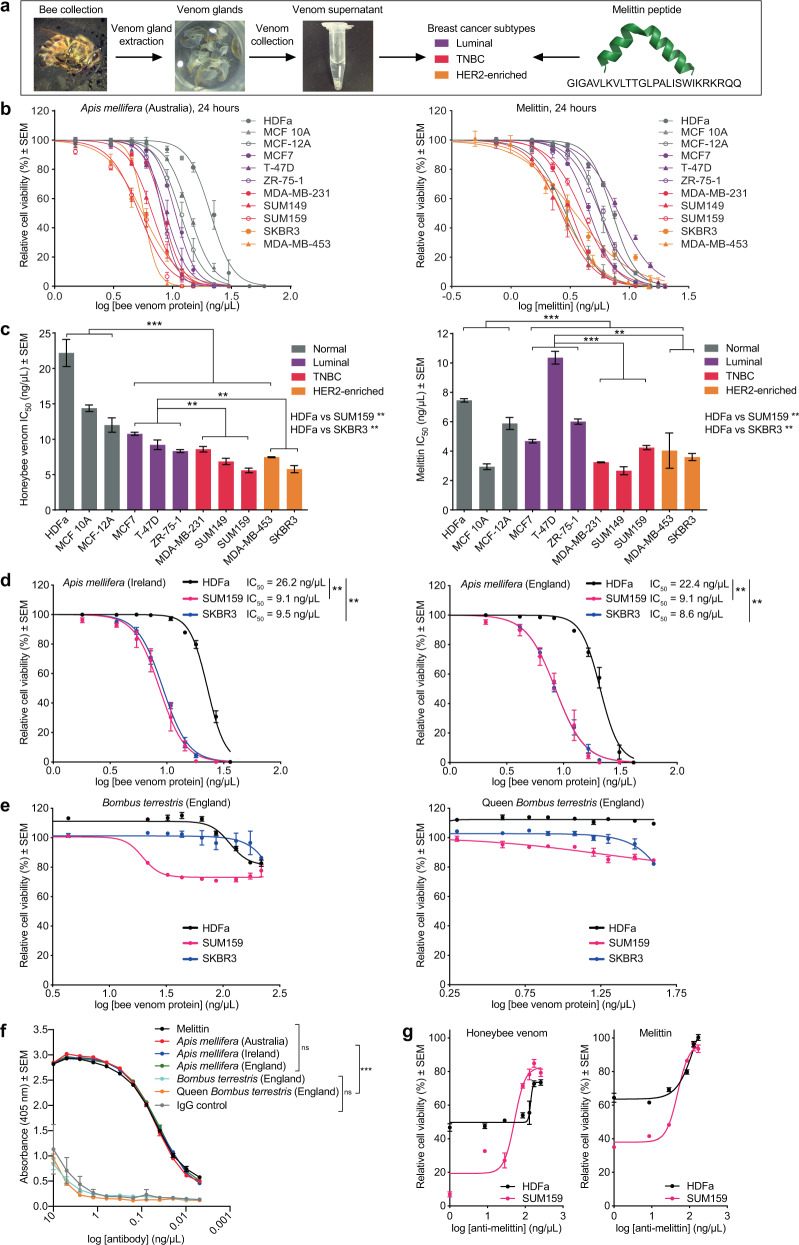
Honeybee venom and melittin specifically reduce breast tumor cell viability. **a** The process of bee venom collection and melittin treatment of breast cancer cells, featuring a honeybee collected in Australia. **b** Cell-viability assays of a panel of human normal and breast cancer cell lines treated with honeybee venom from Australia (left) or melittin (right), with **c** the IC_50_ values (generalized linear models). Cell-viability assays of normal human dermal fibroblasts (HDFa) and breast cancer cell lines (SUM159 and SKBR3) treated with **d** venom from populations of honeybees in Ireland (left) and England (right) (one-way ANOVA), and **e** venom from England worker (left) and queen (right) bumblebees. **f** Absorbance (405 nm) of aqueous solutions of melittin and bee venom assessed by ELISA with the anti-melittin antibody and IgG control (two-way ANOVA). **g** Cell-viability assays in HDFa and SUM159 cells after blocking melittin using the anti-melittin antibody with honeybee venom (left) and melittin (right). Data are represented as mean ± SEM (*n* = 3). Differences were considered significant at *p* < 0.05 (*), *p* < 0.01 (**), and *p* < 0.001 (***). See also Supplementary Fig. 1.

To assess anticancer efficacy and selectivity, venom from both European honeybees collected in Perth, Australia and melittin peptide were evaluated in dose–response assays in a panel of cell lines representative of the intrinsic breast cancer subtypes and in nontransformed cells (Fig. 1a). Honeybee venom showed high anticancer selectivity, with a significantly higher potency in TNBC (e.g., SUM159 and SUM149) and in the HER2-enriched breast cancer cell lines (e.g., MDA-MB-453 and SKBR3), followed by luminal breast cancer cells (including MCF7 and T-47D), with the lowest impact on normal cells (primary dermal fibroblast cells HDFa, and mammary nontransformed MCF 10A and MCF-12A cells) (Fig. 1b, left; Table 1; GLM, Wald Chi-Square = 342, *p* < 0.001, *n* = 33, d*f* = 1). A significant reduction in the half-maximal inhibitory concentration (IC_50_) for both TNBC SUM159 (5.58 ng/μL) and HER2-enriched SKBR3 (5.77 ng/μL) cancer cell lines was observed compared with the normal HDFa cell line (22.17 ng/μL, Fig. 1c, left; one-way ANOVA, *p* < 0.01).

**Table 1:** Half-maximal inhibitory concentrations (IC_50_s) of honeybee venom and melittin.

Cell line	Subtype	Honeybee venom IC_50_ (ng/μL)	Melittin IC_50_ (ng/μL)	Melittin IC_50_ (μM)
*Human*				
HDFa	Normal	22.17 ± 1.91	7.45 ± 0.12	2.62 ± 0.04
MCF 10 A	Normal	14.38 ± 0.47	2.94 ± 0.20	1.03 ± 0.07
MCF-12A	Normal	12.00 ± 1.01	5.88 ± 0.41	2.07 ± 0.14
MCF7	Luminal A	10.77 ± 0.22	4.68 ± 0.12	1.64 ± 0.04
T-47D	Luminal A	9.21 ± 0.69	10.36 ± 0.43	3.64 ± 0.15
ZR-75-1	Luminal A	8.32 ± 0.20	6.01 ± 0.18	2.11 ± 0.06
MDA-MB-231	TNBC, claudin-low	8.58 ± 0.39	3.24 ± 0.03	1.14 ± 0.01
SUM149	TNBC, basal-like	6.86 ± 0.45	2.67 ± 0.27	0.94 ± 0.10
SUM159	TNBC, claudin-low	5.58 ± 0.33	4.24 ± 0.14	1.49 ± 0.05
MDA-MB-453	HER2-enriched	7.46 ± 0.07	4.03 ± 1.20	1.42 ± 0.42
SKBR3	HER2-enriched	5.77 ± 0.51	3.59 ± 0.24	1.26 ± 0.09
*Murine*				
NIH/3T3	Normal	11.7 ± 0.81	4.51 ± 0.18	1.58 ± 0.06
BRCA^−^ B.15	Basal-like	6.42 ± 0.44	2.36 ± 0.19	0.83 ± 0.06
p53^−^ T11	Claudin-low	6.24 ± 0.49	2.08 ± 0.08	0.73 ± 0.03

Data are presented as mean ± SEM in ng/μL or μM (to two decimal places). Experiments were performed in biological triplicates. *TNBC* triple-negative breast cancer, *HER2* human epidermal growth factor receptor 2.

Similarly, melittin was significantly more potent against HER2-enriched breast cancer and TNBC compared to normal cells (Fig. 1b, c, right; Table 1; GLM, Wald Chi-Square = 12.9, *p* < 0.001, *n* = 33, d*f* = 1), with IC_50_ values from 0.94 to 1.49 μM in human TNBC and HER2-enriched breast cancer cells, and 1.03 to 2.62 μM in nontransformed cells. Cell-viability assays of honeybee venom and melittin in murine breast cancer and normal cell lines confirmed enhanced selectivity for aggressive murine tumor cell lines, such as the p53-mutant claudin-low T11 and the BRCA-mutant B.15^37,38^ (Supplementary Fig. 1).

The venom of honeybees from different honeybee populations in Ireland and England reduced the viability of SUM159 and SKBR3 cells significantly more than that of nontransformed HDFa cells (Fig. 1d, one-way ANOVA, *p* < 0.001). We also tested venom from the bumblebee *Bombus terrestris* from England. Samples from both workers and queens elicited minimal cell death in breast cancer cells compared to honeybee venom even at high concentrations of venom (Fig. 1e).

We developed a mouse monoclonal antibody recognizing melittin to assess the relative abundance of melittin in all honeybee and bumblebee venom samples by ELISA. In accordance with the activity studies above, the relative abundance of melittin was not significantly different across all of the honeybee venom samples from different locations (two-way ANOVA, *p* > 0.999). However, melittin concentrations were significantly higher in honeybee samples compared to bumblebee venom and isotype IgG control (Fig. 1f, two-way ANOVA, *p* < 0.001).

The anticancer effects of melittin were confirmed by blocking experiments in vitro, in which we exploited the anti-melittin antibody to rescue cell viability in HDFa and SUM159 cells. Cells were treated with honeybee venom or melittin in combination with increasing concentrations of the anti-melittin antibody. Cell viability was significantly higher when melittin was blocked with the anti-melittin antibody for HDFa and SUM159 cells exposed to honeybee venom or melittin peptide (Fig. 1g, *t* tests, *p* < 0.0001). These data suggest that melittin present in honeybee venom is the most prominent bioactive anticancer compound within all the venoms studied. Honeybee venom collected in Perth, Australia was used for all further experiments.

### Honeybee venom and melittin induce breast cancer cell death

**Fig. 2:**
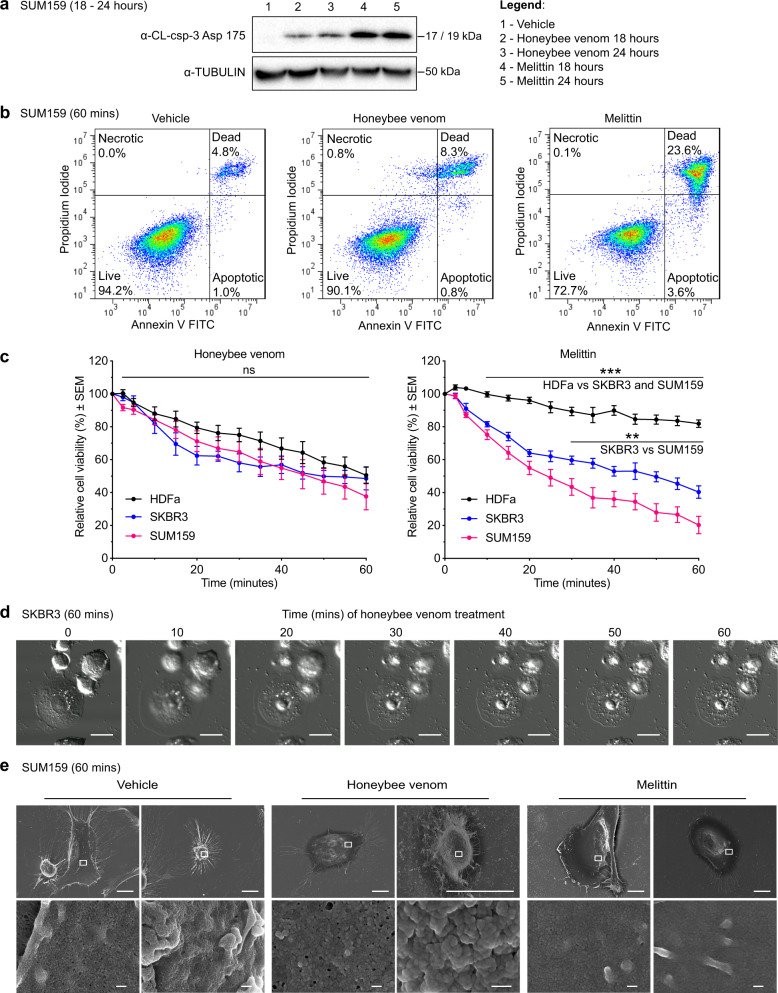
Honeybee venom and melittin induce apoptosis and membrane disruption. **a** Western blot for the detection of cleaved caspase-3 (CL-csp-3) in SUM159 cells treated with vehicle (1), honeybee venom (2–3), and melittin (4–5) for 18 and 24 h. **b** Flow cytometry analysis of SUM159 cells treated with the IC_50_ of honeybee venom (5.58 ng/µL) and the IC_50_ of melittin (4.24 ng/µL) for 1 h. **c** Cell-viability temporal response assays of normal human dermal fibroblasts (HDFa) and breast cancer cells (SUM159 and SKBR3) treated with honeybee venom (left) or melittin (right) over 1 hour (two-way ANOVAs). **d** Live-cell confocal microscopy of SKBR3 cells treated with the IC_50_ of honeybee venom (5.77 ng/µL) over 1 h, with time in minutes post treatment. Scale bars represent 15 µm. **e** Scanning electron microscopy of SUM159 cells treated with the IC_50_ of honeybee venom (5.58 ng/µL) and the IC_50_ of melittin (4.24 ng/µL) over 1 h, with two representative images shown for each treatment group. The white outline in the top images indicates the respective regions of each cell in the bottom images. Scale bars represent 10 µm (top row) and 200 nm (bottom row). Data are represented as mean ± SEM (*n* = 3). Differences were considered significant at *p* < 0.05 (*), *p* < 0.01 (**), and *p* < 0.001 (***). See also Supplementary Figs. 2, 10, and 16.

To examine the mechanism and kinetics of cell death, TNBC cells were treated with the IC_50_ of either honeybee venom or melittin for 18 and 24 h, and processed by a cleaved caspase-3 assay to quantify apoptotic cell death. Immunoblotting confirmed the induction of cleaved caspase-3 in SUM159 cells, with melittin alone inducing a higher level of apoptosis than honeybee venom at both 18 and 24 h post treatment (Fig. 2a, quantification in Supplementary Fig. 2).

To quantify the apoptotic, necrotic, or dead cell populations after treatment, we performed an Annexin V-FITC Apoptosis Detection Assay. SUM159 cells were exposed to vehicle, honeybee venom, or melittin using IC_50_ concentrations and processed by flow cytometry after a 60-min treatment (Fig. 2b). We found significantly more late apoptotic/necrotic cells for the melittin-treated samples (23.6 ± 5.7%) compared to honeybee venom (8.3 ± 1.9%) and vehicle control (4.8 ± 0.4%, two-way ANOVA, *p* < 0.001, mean ± SEM). However, there were no significant differences in the levels of early apoptotic or necrotic cells across all conditions (two-way ANOVA, *p* > 0.05, mean ± SEM). To characterize the kinetics of cell death over shorter times, cell viability was measured for HDFa, SKBR3, and SUM159 cells treated for up to 1 h with IC_50_ concentrations of honeybee venom or melittin (Fig. 2c). Honeybee venom rapidly reduced cell viability, with no significant difference between the normal and cancer cell lines over the hour (two-way ANOVA, *p* = 0.97). In contrast, melittin significantly reduced the viability of both breast cancer cell lines compared to the normal cells from 10 min onward, and SUM159 significantly more than SKBR3 from 30 min onward (two-way ANOVA, *p* < 0.0001).

Live-cell confocal microscopy (Fig. 2d) and scanning electron microscopy (Fig. 2e) in SKBR3 and SUM159 cells illustrated a rapid disruption and shrinking of the plasma membrane with honeybee venom and melittin treatment relative to vehicle treatment over 10 to 60 min.

### RGD enhances the breast cancer targeting of melittin

**Fig. 3:**
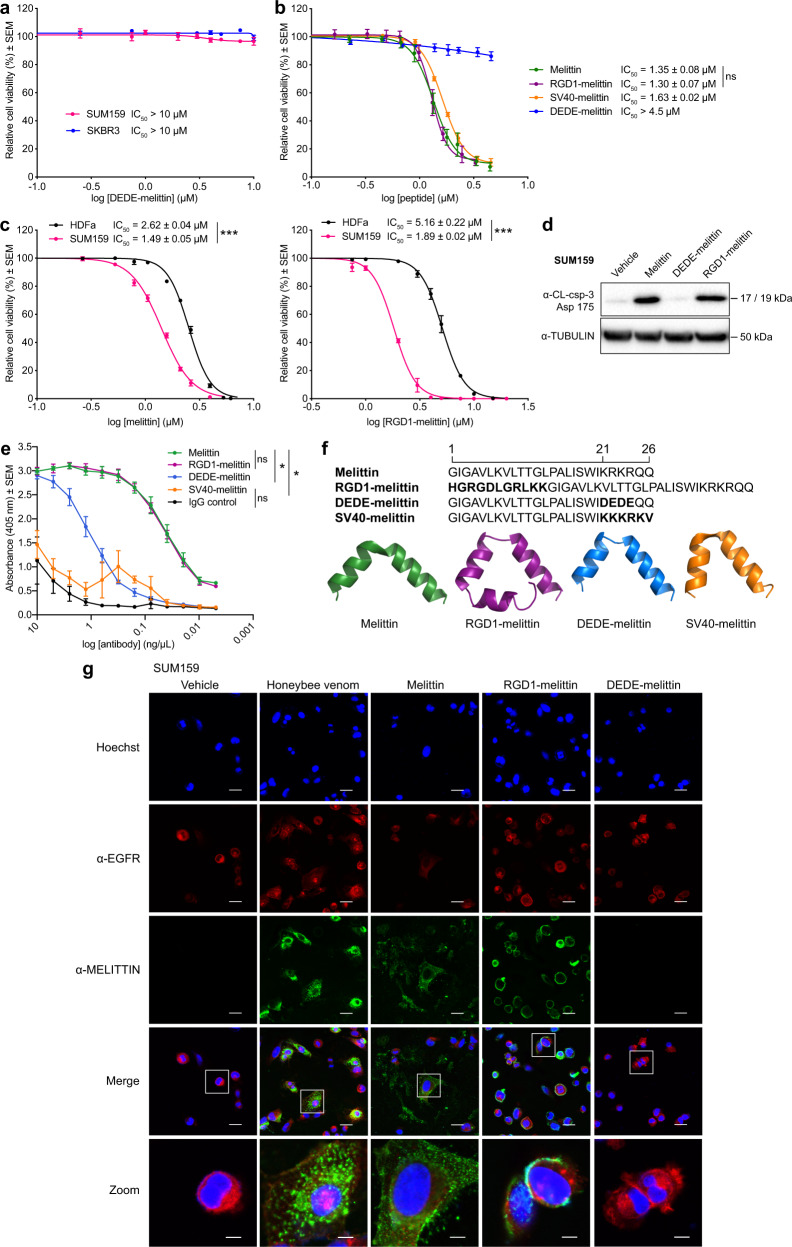
Engineering melittin with an RGD motif enhances breast cancer selectivity. **a** Cell-viability assays of TNBC (SUM159) and HER2-enriched breast cancer (SKBR3) cells treated with DEDE-melittin for 24 h. **b** Cell-viability assays of T11 cells treated with melittin, RGD1-melittin, SV40-melittin, and DEDE-melittin for 24 h (*t* test). **c** Cell-viability assays of normal human dermal fibroblasts (HDFa) and SUM159 treated with melittin (left) and RGD1-melittin (right) for 24 h (*t* tests). **d** Western blot for the detection of cleaved caspase-3 (CL-csp-3) in lysates from SUM159 cells treated with vehicle, melittin, DEDE-melittin, or RGD1-melittin for 24 h. **e** Absorbance (405 nm) of aqueous solutions of melittin, RGD1-melittin, DEDE-melittin, and SV40-melittin subjected to an ELISA with the anti-melittin antibody (two-way ANOVA). **f** The amino-acid sequence and top predicted 3D model of melittin (green), RGD1-melittin (purple), DEDE-melittin (blue), and SV40-melittin (orange). **g** Immunofluorescence images of SUM159 treated with vehicle, honeybee venom, melittin, RGD1-melittin, or DEDE-melittin for 30 min. In blue: cell nuclei, in red: anti-EGFR, and in green: anti-melittin. The white outlines in the merged images indicate the respective regions in the zoomed images. Scale bars represent 25 µm, and 6.25 µm for the zoomed images. Data are represented as mean ± SEM (*n* = 3). Differences were considered significant at *p* < 0.05 (*), *p* < 0.01 (**), and *p* < 0.001 (***). See also Supplementary Figs. 3 and 10.

The C-terminus of melittin forms a positively charged α-helix that has been proposed to mediate binding to the negatively charged plasma membrane, inducing subsequent pore formation and cell lysis^39–41^. Previous studies have shown that truncating this positively charged C-terminus significantly reduces melittin binding to phospholipid bilayers compared to wild-type melittin^39,42^. To assess the functional role of the positive (K_21_RKR_24_) sequence in the C-terminus of melittin, we designed a negatively charged melittin peptide (D_21_EDE_24_-melittin). These negative residues were predicted to disrupt the binding of melittin with the plasma membrane. We found that DEDE-melittin elicited no measurable signs of anticancer activity in any of the cell lines tested (Fig. 3a, b). Importantly, the anticancer activity of DEDE-melittin was rescued with a positively charged sequence (K_21_KKRKV_26_) present in the Simian Virus 40 (SV40) large T antigen (peptide SV40-melittin) possessing cell-penetrating capacity^43^ (Fig. 3b). Similarly, grafting a larger positively charged TAT sequence (transactivator of transcription, derived from HIV-1)^43^ in the C-terminus of melittin also restored the activity of DEDE-melittin (peptide TAT-melittin; Supplementary Fig. 3). However, the potency of melittin and SV40-melittin was greater than TAT-melittin, which could be due to the larger size of TAT. These data demonstrate that residues required for melittin activity include those residing in the C-terminal α-helix, comprising several key positively charged residues necessary for interaction with the plasma membrane.

To enhance cancer cell selectivity, we generated a bifunctional melittin peptide by engineering an N-terminal alpha-helical RGD peptide motif (RGD1-melittin, derived from TGF-β3, sequence HGRGDLGRLKK), which interacts with αvβ6 and αvβ3 integrins overexpressed on breast cancer cell membranes and tumor-associated vasculature^44–46^. When engineered with bioactive peptides, RGD motifs enhance targeting to breast cancer cells^47^. The IC_50_ of RGD1-melittin was not significantly different compared to parental melittin in T11 cells, indicating that the potency was not affected by the RGD motif (Fig. 3b, *t* test, *p* = 0.652). Taking the ratios of the IC_50_s of HDFa/SUM159 for RGD1-melittin (2.73 ± 0.14) compared to melittin (1.76 ± 0.04), the RGD motif significantly increased the therapeutic window between the normal and TNBC cell lines, confirming enhanced cancer cell selectivity conferred by RGD (Fig. 3c, *t* test, *p* < 0.01, mean ± SEM). Induction of apoptosis in the SUM159 TNBC cells treated with melittin, DEDE-melittin, and RGD1-melittin for 24 h confirmed the anticancer activity of both melittin and RGD1-melittin, but not DEDE-melittin (Fig. 3d).

Consistent with the anticancer activity of melittin and RGD1-melittin, we found that the interaction between the anti-melittin antibody and melittin was not significantly different from that with RGD1-melittin (Fig. 3e, two-way ANOVA, *p* > 0.999), but was significantly different from DEDE-melittin and SV40-melittin (two-way ANOVA, *p* < 0.05), with the absorbance of SV40-melittin not significantly different from IgG control (two-way ANOVA, *p* > 0.1). These data suggested that our monoclonal anti-melittin antibody recognizes a conformational epitope that is not disrupted by the engineering of an N-terminal targeting peptide.

Modeling studies indicated that the conformation of the melittin portion of the engineered peptides was not disrupted by either the C-terminal mutations or the N-terminal addition of the RGD motif (Fig. 3f). Each peptide retained the characteristic bent alpha-helix structure potentially facilitating the formation of pores^4^, suggesting that differences in anticancer activity between the mutants are due to electrostatic interactions with the membrane and not gross changes in peptide structure.

We next exploited the anti-melittin antibody to detect the subcellular localization of the active peptides by immunofluorescence in TNBC SUM159 cells treated for 30 min with vehicle, honeybee venom, melittin, RGD1-melittin, or DEDE-melittin at IC_50_ concentrations (Fig. 3g). Independently of whether cells were exposed to honeybee venom, melittin, or RGD1-melittin, melittin predominantly localized to the plasma membrane of cells overexpressing EGFR, with a degree of intracellular staining in honeybee venom and melittin-treated cells, potentially due to membrane disruption and the formation of endosomes as reported elsewhere^25,48^. Moreover, the pattern of staining of RGD1-melittin appeared distinctively targeted to the plasma membrane alone, which would be in keeping with enhanced selectivity of the targeted peptide for tumor cell surface moieties. We observed a lack of reactivity of the melittin antibody in DEDE-melittin-treated cells. In summary, these results reveal that while the RGD motif enhances the targeting of melittin to breast cancer cell membranes, the C-terminal positive motif seems essential for anticancer activity.

### Honeybee venom and melittin suppress RTK phosphorylation

**Fig. 4:**
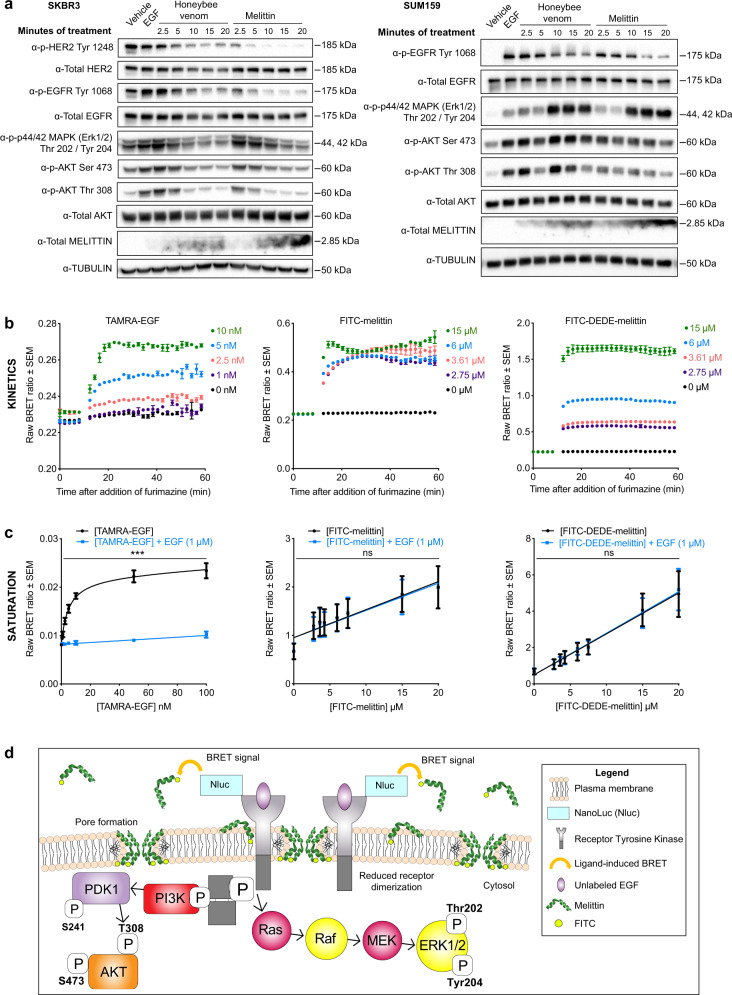
Honeybee venom and melittin suppress the phosphorylation of EGFR and HER2. **a** Phosphorylation kinetics of HER2, EGFR, and downstream MAPK and Akt pathways after treatment with honeybee venom and melittin in SKBR3 (left) and SUM159 (right) breast cancer cells, assessed by immunoblotting. **b** Bioluminescence resonance energy transfer (BRET) kinetic analysis of TAMRA-EGF, FITC-melittin, and FITC–DEDE-melittin interaction with NanoLuc-EGFR in HEK293FT cells. The peptides were added after the cells were equilibrated in the reader with the NanoLuc substrate furimazine for 5 min. **c** Saturation-binding analysis of increasing concentrations of TAMRA-EGF, FITC-melittin, and FITC–DEDE-melittin in HEK293FT cells transfected with NanoLuc-EGFR in the presence or absence of unlabeled EGF (1 µM). Data are expressed as raw BRET ratios and represented as mean ± SEM (*n* = 3, two-way ANOVA). **d** Proposed model of action of melittin interfering with the dimerization and phosphorylation of RTKs in the plasma membrane. Differences were considered significant at *p* < 0.05 (*), *p* < 0.01 (**), and *p* < 0.001 (***). See also Supplementary Figs. 4–6 and 11–15.

We subsequently investigated if both honeybee venom and melittin disrupt RTK-associated signaling pathways by blocking the ligand-dependent activation of EGFR and HER2 in breast carcinoma cells. To assess this, we conducted immunoblotting analysis on SKBR3 (HER2^+^ and EGFR^+^) and SUM159 (EGFR^+^) extracts of cells exposed to EGF and treated with the IC_50_ of honeybee venom or melittin from 2.5 to 20 min (Fig. 4a). Both honeybee venom and melittin downregulated the phosphorylation of the RTKs and modulated the associated PI3K-/Akt and MAPK signaling pathways in a time-dependent manner.

Honeybee venom and melittin treatment in SKBR3 cells strongly downregulated p-HER2 (Tyr1248), p-EGFR (Tyr1068), p-p44/42 MAPK (Thr202/Tyr204), p-Akt (Ser473 and Thr308), p-SAPK/JNK (Thr183/Tyr185), and p-p38 MAPK (Thr180/Tyr182) from 5 min onward (Fig. 4a, left; Supplementary Fig. 4), with a slight decrease in total HER2, EGFR, and Akt protein only after 10 min of honeybee venom treatment, which could relate to endosome-mediated receptor degradation^25^. In SUM159, p-EGFR (Tyr1068) was strongly downregulated by honeybee venom and melittin from 10 to 20 min. Treating SUM159 with melittin also suppressed p-Akt (Ser473 and Thr308) at all time points, yet upregulated p-p44/42 MAPK (Thr202/Tyr204), p-SAPK/JNK (Thr183/Tyr185), and p-p38 MAPK (Thr180/Tyr182) from 10 to 20 min, whereas honeybee venom upregulated p-p44/42 MAPK (Thr202/Tyr204) and p-Akt (Ser473 and Thr308) from 10 to 20 min (Fig. 4a, right; Supplementary Fig. 4). The MAPK and Akt pathways may have been upregulated in SUM159 cells due to the release of a negative regulatory feedback loop that triggers ERK signaling to protect the cells from apoptotic cell death^8,49^. The anti-melittin antibody indicated an increasing amount of melittin present in the lysates of both cell lines over time, with a stronger signal for the melittin treatment compared to honeybee venom in both cell lines.

To characterize the effects on signaling pathways in another TNBC model, we conducted immunoblotting on MDA-MB-231 cells, in which EGF treatment phosphorylated EGFR and induced EGFR expression (Supplementary Fig. 4). Melittin reduced the phosphorylation of EGFR and MAPK, downregulating major oncogenic proliferation pathways. Unlike SUM159 cells, EGFR stimulation by EGF did not correlate with an increase in phosphorylation in p-Akt, potentially due to disengagement between EGFR signaling and Akt pathways. Other growth factor receptors, such as VEGFR1, may mediate the activation of these pathways^50,51^. While melittin previously inhibited JAK2/STAT3 signaling in ovarian cancer^12^, no modulatory effects were observed on JAK/STAT pathway inhibitors in SUM159 cells after a 60-min treatment with honeybee venom or melittin (Supplementary Fig. 5).

Considering that TNBC and HER2-enriched breast carcinoma cells are highly dependent on the activation of EGFR and HER2, we performed bioluminescence resonance energy transfer (BRET) experiments to determine whether melittin interfered with the binding of EGF to EGFR, leading to the observed suppressed growth factor receptor phosphorylation. The NanoLuc reporter was used as the bioluminescent donor molecule and genetically fused to EGFR^52,53^. Kinetic and saturation BRET experiments were used to monitor the proximity of NanoLuc-EGFR with the fluorescently tagged acceptor molecules TAMRA-EGF (positive control), FITC-melittin, and FITC–DEDE-melittin (negative control) in HEK293FT cells transfected with NanoLuc-EGFR. Transfer of energy from the bioluminescent donor to the fluorescent acceptor occurs over distances less than 10 nm, and is indicative of interactions between the tagged molecules of interest^54^. The BRET signal is determined by monitoring the ratio of light emission from the acceptor over the emission from the donor.

A range of concentrations of each peptide was selected, including the IC_50_ of FITC-melittin, with the corresponding molar concentrations of FITC–DEDE-melittin. We found that the BRET signal increased in a dose-dependent manner for TAMRA-EGF and FITC–DEDE-melittin, and to a lesser extent for FITC-melittin (Fig. 4b). FITC–DEDE-melittin displayed much higher BRET ratios than FITC-melittin at the same concentrations, as well as reaching maximal BRET ratios at each dose very rapidly. A nonspecific peptide designed against the Engrailed 1 (EN1) transcription factor^55^ (FITC–EN1-mutant) exhibited similar BRET ratios and kinetics to FITC–DEDE-melittin (Supplementary Fig. 6), indicating that further experiments were required to ascertain the specificity of the binding interactions with EGFR.

To determine the specificity of melittin binding to EGFR at the EGF-binding site, we conducted saturation BRET assays to assess competition of EGF with each of the peptides binding to NanoLuc-EGFR (Fig. 4c). While the binding of TAMRA-EGF to NanoLuc-EGFR was saturable and significantly reduced in the presence of 1 µM EGF (two-way ANOVA, *p* < 0.0001), the BRET signals of FITC melittin and FITC–DEDE-melittin were not saturable and not significantly different with or without 1 µM EGF (two-way ANOVA, *p* > 0.999), suggesting that neither melittin nor DEDE-melittin bound at the EGF-binding site.

Our data support the notion that melittin becomes incorporated into the plasma membrane of cancer cells via a charged sequence present in the C-terminus, inducing plasma membrane remodeling and disruption. BRET data indicate that melittin may be positioned at a distance within 10 nm from RTKs without interfering with the endogenous growth factor-binding site (Fig. 4d).

### Melittin sensitizes TNBC to docetaxel treatment in vivo

**Fig. 5:**
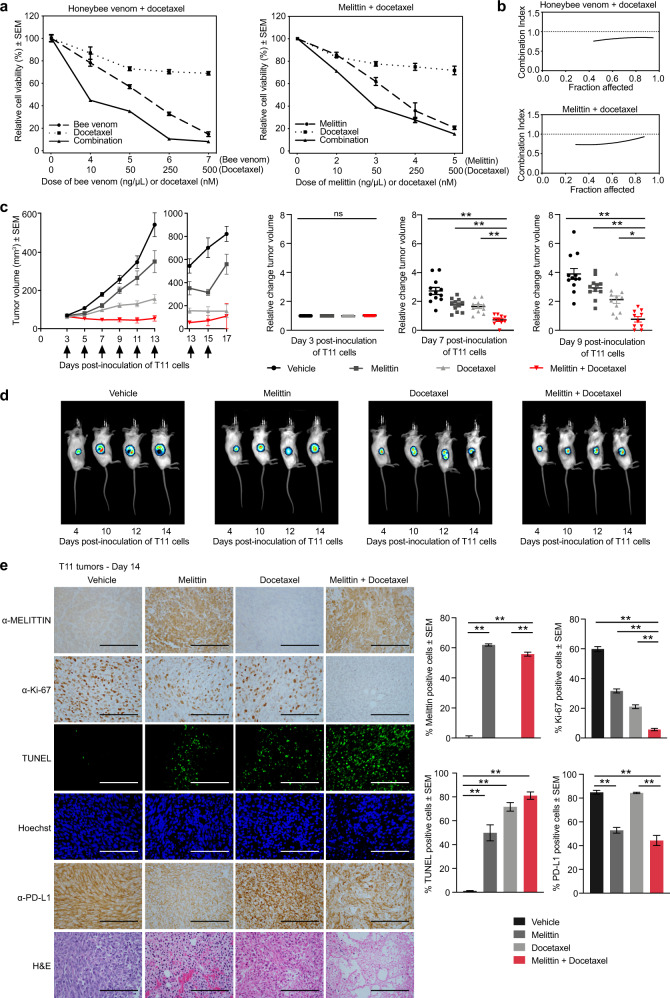
Melittin sensitizes highly aggressive TNBC tumors to docetaxel treatment in vivo. **a** Cell-viability assays of T11 cells treated with honeybee venom and melittin alone and in combination with docetaxel for 24 h. Representative plots of the combination treatments are presented (*n* = 3). **b** Combination index graphs obtained for different fractions of cells affected in each combination, calculated using CompuSyn software. **c** Tumor volumes of mice treated intratumorally with vehicle, 5 mg/kg melittin, 7 mg/kg docetaxel, and 5 mg/kg melittin + 7 mg/kg docetaxel. Arrows indicate the treatment days. Corresponding scatter plots of relative change in tumor volumes at days 3, 7, and 9 are indicated (one-way ANOVA, *n* = 12). **d** Representative bioluminescence imaging (BLI) of T11-luciferase tumors in mice at days 4, 10, 12, and 14 post inoculation of the cells. **e** Representative images of immunohistochemistry and immunofluorescence in tumor biopsies from mice extracted on day 14 post T11 inoculation stained with anti-melittin, anti-Ki-67, TUNEL assay, Hoechst, anti-PD-L1, and H&E (one-way ANOVA, *n* = 8). Scale bars represent 100 µm. Data are represented as mean ± SEM. Differences were considered significant at *p* < 0.05 (*), *p* < 0.01 (**), and *p* < 0.001 (***). See also Supplementary Figs. 7–9.

We next tested for potential synergies between melittin and chemotherapeutic agents to increase breast cancer cell death. The murine p53^−^ TNBC cell line T11 was treated with docetaxel in combination with either honeybee venom or melittin, and cell-viability assays were conducted to determine the combination index (CI) between the treatments^56^ (Fig. 5a). We observed CIs < 1 for all the concentrations tested, indicating strong synergistic interactions (Fig. 5b). Synergisms were also observed with cisplatin, an agent used to treat TNBCs in the clinic (Supplementary Fig. 7). The T11 xenograft model was used for in vivo experiments because it demonstrated the most favorable in vitro drug interaction between melittin and docetaxel across multiple cell lines tested (Supplementary Fig. 8), and it has an intact immune system enabling the immune response to melittin to be assessed.

To investigate the efficacy of the combination of melittin and docetaxel in reducing TNBC growth, we performed in vivo experiments by transplanting T11 cells in BALB/c mice. This allograft model recapitulates highly aggressive, TNBC claudin-low disease in mice with an intact immune system^38,57,58^. Three days after the generation of T11 tumors (~50 mm^3^), mice were randomized into four groups (*n* = 12 mice/group) and treated intratumorally with vehicle, melittin (5 mg/kg), docetaxel (7 mg/kg), or a combination of melittin (5 mg/kg) and docetaxel (7 mg/kg). Mice were treated every 2 days from day 3, with 7 treatments in total. We found that for the combination treatment, tumor control was superior compared to either treatment alone or vehicle, particularly on days 7 and 9 post inoculation of T11 cells, with the combination achieving a significant reduction in tumor volume (Fig. 5c, one-way ANOVA, *p* < 0.001). This suggests that tumors resistant to docetaxel could be rendered sensitive by the addition of melittin. We validated these studies by bioluminescence imaging (BLI) to noninvasively track changes in in vivo tumor growth in T11 cells tagged with a *luciferase*-containing construct (Fig. 5d). Here again, we found an improved tumor control for the docetaxel and melittin combination treatment at days 10, 12, and 14 compared to all other groups.

The therapeutic effects of melittin and docetaxel were validated in tumor tissues at day 14 post inoculation of T11 cells by immunohistochemistry and immunofluorescence (Fig. 5e). The anti-melittin antibody confirmed the intra-tumoral localization of melittin-positive cells in both the melittin (61.9 ± 0.7%) and the combination treatment groups (55.8 ± 1.3%), but not in vehicle control (one-way ANOVA, *p* < 0.01, mean ± SEM). A significant reduction in tumor cell proliferation (as assessed by Ki-67 expression) was found in the tumors treated with the combination of melittin and docetaxel (5.7 ± 0.8%) relative to vehicle (59.8 ± 1.7%), compared to either melittin (31.7 ± 1.3%) or docetaxel alone (21.0 ± 1.3%, one-way ANOVA, *p* < 0.01, mean ± SEM). TUNEL staining confirmed a significantly higher DNA fragmentation and apoptosis induction in the combination group (81.0 ± 3.1%) compared to vehicle (1.0 ± 0.4%, one-way ANOVA, *p* < 0.01, mean ± SEM).

The immune-checkpoint protein programmed death ligand-1 (PD-L1) reduces the functionality of activated T cells. Consequently, immune-checkpoint blockades in combination with chemotherapy prevent T-cell PD-L1 recognition, preventing this adaptive immune resistance in TNBC, and thereby increasing therapeutic efficacy over chemotherapy alone^59^. In contrast to docetaxel alone (84.3 ± 0.6%) that did not affect the levels of PD-L1 in the tumors, we found that melittin significantly reduced PD-L1 expression in tumors when used alone (52.9 ± 2.4%) or with the combination treatment (44.3 ± 4.2%) compared to vehicle (84.9 ± 1.6%, one-way ANOVA, *p* < 0.01, mean ± SEM). In summary, these studies support the notion that melittin sensitizes T11 cells to docetaxel treatment, and that melittin could help attenuate the expression of immune-checkpoint proteins, consequently improving antitumoral immune responses.

Next, we performed immunohistochemistry in the treated T11 tumors to detect p-HER2 (Tyr1248) and p-EGFR (Tyr1068) (Supplementary Fig. 9). The expression of EGFR was moderately but significantly reduced by the melittin and docetaxel combination (75.8 ± 6.4%) compared to vehicle (100.0 ± 9.1%, one-way ANOVA, *p* < 0.05, mean ± SEM). The expression of HER2 was not significantly different across all treatment groups (one-way ANOVA, *p* = 0.1536). For p-EGFR (Tyr1068), the phosphorylation was reduced to a significantly lower level by the melittin and docetaxel combination treatment (9.0 ± 2.4%) compared to vehicle (100.0 ± 8.1%, one-way ANOVA, *p* < 0.0001, mean ± SEM). The levels of p-HER2 (Tyr1248) were also reduced to a significantly lower level in the melittin and docetaxel combination treatment (50.3 ± 7.8%) compared to vehicle (100.0 ± 5.6%, one-way ANOVA, *p* < 0.0001, mean ± SEM). The decrease in EGFR and HER2 phosphorylation in vivo after melittin treatment is consistent with the observed effects of melittin in reducing the phosphorylation of these RTKs in SKBR3, SUM159, and MDA-MB-231 cells (Fig. 4a; Supplementary Fig. 4).

## Discussion

Apitherapy is an emerging field with the potential to impact the economic aspects of cancer research globally, particularly in under-resourced communities. To date, however, studies are yet to fully investigate the molecular mechanism of action of honeybee venom and melittin, and their consequent optimum usage in the oncology arena is yet to be comprehensively investigated, particularly for the treatment of breast cancer, the most commonly occurring cancer in women worldwide^2^. TNBCs and HER2-enriched tumors are highly aggressive breast cancer subtypes. TNBC is associated with the highest mortality and, despite frequent EGFR expression, commonly displays resistance to anti-EGFR therapies with high dependence on PI3K/Akt signaling for proliferation, survival, and chemotherapy resistance^34^.

Anti-HER2 therapies have substantially improved long-term survival in early-stage HER2-positive cancers, but the majority of late-stage patients eventually develop resistance and succumb to the disease^33,35,36^. Not only did we demonstrate selectivity of honeybee venom and melittin for malignant cells, but we also revealed higher potencies for these aggressive types of breast cancer.

Here, we show that honeybee venom and melittin suppress the ligand-induced phosphorylation of EGFR and HER2, dynamically modulating downstream signaling pathways in breast cancer cells. We propose that melittin directly or indirectly inhibits RTK dimerization. Melittin may also enter the cell to directly or indirectly modulate downstream signaling pathways^25,60^. Previous work has shown that melittin can be targeted to HER2-overexpressing cell lines using immunoliposomes bearing trastuzumab^61^. Here, we demonstrate that melittin alone selectively targets HER2- and EGFR-overexpressing breast cancer cells. Interestingly, melittin was more potently toxic to breast cancer cells compared to honeybee venom, warranting further investigation.

In our study, we focused on the cell lines SUM159 and SKBR3. SUM159 is a TNBC cell line that expresses the EGFR gene product and harbors missense mutations in PI3KCA (H1047L) and in HRAS (G12D)^62,63^. In contrast, SUM159 is KRAS, NRAS, BRAF, PTEN, and MAP2K4 wild type and negative for AKT1 activation and AKT2 and AKT3 amplification^63^. SKBR3 is a HER2-enriched breast cancer cell line that overexpresses the HER2 gene product^64^, and is KRAS, HRAS, NRAS, BRAF, PTEN, PI3KCA, and MAP2K4 wild type^63,65,66^, and also negative for AKT1 activation, and AKT2 and AKT3 amplification^63^. Taking these molecular characteristics into consideration, the EGFR downstream signaling pathways are not constitutively activated in SUM159 cells, despite the existing mutations in HRAS and PI3KCA, as these are not sufficient to basally activate these pathways^67^.

We report a potent and synergistic antitumor response with melittin and docetaxel in a highly aggressive TNBC model in vivo. This highlights the potential for melittin for use in combination therapies to potentially increase the efficacy and/or reduce the dose of cytotoxic agents, enabling more cost-effective treatments with potentially less side effects to be delivered. Melittin also reduced the levels of the PD-L1 immune-checkpoint protein involved in immune evasion. Melittin could therefore decrease the immune-suppressive effects of the tumor microenvironment, which are prevalent in TNBCs in the presence of chemotherapy. This adds to data from previous reports showing that melittin can also reduce the tumor-promoting M2-like tumor-associated macrophage population in the tumor microenvironment in a lung carcinoma model^68^. We hypothesize that in our in vivo T11 model, EGFR and HER2 signaling may modulate PD-L1 expression in tumor cells. According to previous immunohistochemical studies, PD-L1 has the highest expression in TNBC tumors, followed by HER2-enriched tumors^69–72^, and PD-L1 expression is associated with poor survival^69^. In basal-like breast cancers, the absence of the protein ALIX was shown to correlate with EGFR activation, impairing exosome biogenesis^73^. PD-L1 is secreted via exosomes in an ALIX-dependent manner, such that exosome impairment increases PD-L1 on the cell membrane. ALIX downregulation promotes tumor survival through enhancement of EGFR activation, and through PD-L1 membrane accumulation, leading to immunosuppression^73^. In HER2-enriched breast cancer, the crosstalk between HER2 and PD-L1 is poorly understood^74^. However, in HER2-positive breast cancer cells cocultured with human peripheral blood mononuclear cells and in a mouse model, trastuzumab anti-HER2 therapy resulted in upregulation of PD-L1^75,76^. Hence the incorporation of melittin with trastuzumab could abrogate this immune-suppressive response.

The selectivity of melittin for HER2-driven tumors also makes a further case for combination with HER2-targeted agents, including monoclonal antibodies, trastuzumab-emtansine, and other antibody–drug conjugates where the membrane-disrupting properties of melittin could enhance the internalization kinetics of the cytotoxic payload. Our work also reveals new opportunities to modify specific regions of melittin to further increase the effectiveness and targeted specificity for malignant cells. Engineered targeted peptides, such as RGD1-melittin, could be delivered intravenously to enable a more selective homing and uptake into tumor cells. Melittin could also be delivered through targeted nanoparticle approaches, such as those previously reported with “nanobees”^77,78^. Linking melittin with toxins or prodrugs could also be exploited, as reported with uPA-cleavable melittin fusions^79^. Future studies to formally assess toxicities and maximum tolerated doses of these peptides will be required prior to human trials.

Honeybee venom is available globally and offers cost-effective and easily accessible treatment options in remote or less-developed regions. Further research will be required to assess whether the venom of some genotypes of bees has more potent or specific anticancer activities, which could then be exploited. Beyond breast cancer, tumors overexpressing EGFR include lung, glioblastoma, and colorectal cancers^80^, and tumors that can overexpress HER2 include gastric, ovarian, endometrial, bladder, lung, colon, and head and neck cancers^81^. Overall, our results could be leveraged to aid the development of new therapeutic modalities for many cancer types associated with frequent drug resistance and poor prognosis.

## Methods

### Chemical reagents and antibodies

All peptides were purchased from China Peptides Corporation, Ltd. A fluorescent fluorescein isothiocyanate (FITC) tag was conjugated to the N terminus of FITC-melittin, SV40-melittin, TAT-melittin, and EN1-mutant. CellTiter-Glo 2.0 from the Luminescent Cell Viability Assay, NanoLuc-EGFR, FuGENE, and furimazine were all obtained from Promega. TAMRA-EGF was obtained from Invitrogen (Thermo Fisher Scientific). Docetaxel (Cat. No. D-1000) was obtained from LC Laboratories. The monoclonal antibody to α-Tubulin (1:5000, Cat. No. T5168), Hoechst (1:5000, Cat. No. 94403), and human EGF (Cat. No. E9644) were obtained from Sigma-Aldrich. Mouse EGF (Cat. No. 315-09) was obtained from Peprotech. Antibodies against phospho-HER2 (Tyr1248) (immunoblotting: 1:1000, immunohistochemistry: 1:100, Cat. No. 2247), phospho-EGFR (Tyr1068) (immunoblotting: 1:1000, Cat. No. 2234; immunohistochemistry: 1:350, Cat. No. 3777, clone D7A5), phospho-p44/42 MAPK (Erk1/2) (Thr202/Tyr204) (1:2000, Cat. No. 4370), phospho-Akt (Ser473) (1:2000, Cat. No. 4060), phospho-Akt (Thr308) (1:1000, Cat. No. 13038), phospho-SAPK/JNK (Thr183/Tyr185) (1:1000, Cat. No. 4668), phospho-p38 MAPK (Thr180/Tyr182) (1:1000, Cat. No. 4511), Total AKT (1:1000, Cat. Nos. 9272 and 4685), Cleaved Caspase-3 (Asp175) (1:1000, Cat. No. 9661), Ki-67 (1:400, Cat. No. 9449), the Jak/Stat Pathway Inhibitors Antibody Sampler Kit (1:1000, Cat. No. 8343), and the secondary anti-mouse IgG, HRP-linked antibody (1:10,000, Cat. No. 7076), and anti-rabbit IgG, HRP-linked antibody (1:10,000, Cat. No. 7074) were manufactured by Cell Signaling Technology. Monoclonal antibodies against ErbB2 (immunoblotting: 1:1000, immunohistochemistry: 1:100, Cat. No. ab8054, clone CB11), EGFR (immunoblotting: 1:5000, immunohistochemistry: 1:100, Cat. No. ab52894, clone EP38Y), and PD-L1 [PD-L1/2746] (1:100, Cat. No. ab238697) were manufactured by Abcam. The Alexa Fluor 488 goat anti-mouse (1:500, Cat. No. A11001) and Alexa Fluor 594 goat anti-rabbit (1:500, Cat. No. A11012) secondary antibodies were obtained from Thermo Fisher Scientific. The polyclonal goat anti-mouse IgG γ-chain-specific secondary antibody (ELISA: 1:1000, Cat. No. AP503P) was obtained from Millipore. The mouse monoclonal IgG antibody specific to human IL-12 (28/00 8C1-6) used as the control antibody for the ELISA experiments, and the mouse monoclonal IgG antibody specific to melittin (ELISA: 1:350, clone 3B9) were produced at the Monoclonal Antibody Facility at the Harry Perkins Institute of Medical Research. The TUNEL assay (In Situ Cell Death Detection Kit) was obtained from Roche.

### Bee venom collection

The venom was collected using workers or queens from several different populations of Apid bees. Venom samples collected from European honeybees (*Apis mellifera*) and buff-tailed bumblebees (*Bombus terrestris audax*) originated from Perth (Australia), Dublin (Ireland), and London (England). Honeybee venom was collected from 30 workers of each of three different colonies from an apiary or farm as described. Honeybee venom from Australia was collected from an apiary maintained by the Centre for Integrative Bee Research (CIBER), located at the University of Western Australia (UWA: −31.980151, 115.817919). Honeybee venom from Ireland was collected from one colony at an apiary at Trinity College Dublin (53.343933, −6.254635), and the other two colonies from farms near Glasnevin (53.383245, −6.276333) and Blanchardstown (53.384220, −6.375979). Honeybee and bumblebee venom from England was collected at the Royal Holloway University of London (51.425626, −0.562987). Bumblebee venom was collected from 20 workers from each of 2 commercially purchased colonies, with the single-queen bumblebees from each of these two colonies used for the collection of queen bumblebee venom. Independent biological master mixes were prepared by keeping the venom from different colonies separate, with the venom of 312 bees collected in total.

Glandular venom was collected by manual dissection. Bees were captured near the entrance of the hive for honeybees, or directly from the colony for bumblebees, and anesthetized with carbon dioxide and chilled on ice. The sting apparatus was dissected from each individual; then the venom gland removed and placed in phosphate-buffered saline (PBS). The glands were pierced with a Terumo Needle (25 G × 5/8) and centrifuged (13,000*g*, 10 min, 4 °C), and the supernatant collected, containing venom in liquid suspension. The protein concentration of each master mix was quantified with a Detergent Compatible Protein Assay (Bio-Rad), measuring absorbance at 750 nm with a Millennium Science BioTek PowerWave XS2 (Gen 5 1.11 Software, Version 1.11.5). Each master mix was then aliquoted and stored at −80 °C.

### Cell lines and culture conditions

All cell lines were purchased from the American Type Culture Collection (Manassas, VA, USA), except for HEK293FT cells that were purchased from Invitrogen (Thermo Fisher Scientific, Victoria, Australia), SUM149 and SUM159 that were obtained from Asterand Bioscience (Detroit, MI, USA), and T11 and B.15 cells that were kindly provided by Charles Perou and Lyuba Varticovski from the University of North Carolina at Chapel Hill and National Institutes of Health, respectively. T11 and B.15 are very well-characterized cell lines^37,38^.

Cells were incubated at 37 °C and 5% CO_2_ and supplemented with 1% antibiotic–antimycotic. HDFa (normal primary adult human dermal fibroblast) cells were cultured in DMEM with 10% fetal bovine serum (FBS). MCF 10A and MCF-12A (human mammary immortalized epithelial cells, nontransformed) were maintained in DMEM/F-12 with supplements (5% fetal horse serum, 20 ng/mL epidermal growth factor, 10 μg/μL insulin, 100 ng/mL cholera toxin, and 500 ng/mL hydrocortisone). NIH/3T3 (murine embryonic fibroblast) cells were maintained in DMEM with 10% FBS. HEK293FT (human embryonic kidney 293 cells stably expressing the SV40 large T antigen) was cultured in DMEM with 10% FBS and supplements (1% glutamine and 0.4 mg/mL G418 Geneticin, Gibco). MCF7 (human luminal A breast cancer) was maintained in MEM α with 10% FBS and supplements (1% each of sodium pyruvate, sodium bicarbonate, and nonessential amino acids). T-47D and ZR-75-1 (both human luminal A breast cancer) were cultured in RPMI with 10% FBS. MDA-MB-231 (human claudin-low breast cancer) was cultured in DMEM with 10% FBS. SUM149 (human basal-like breast cancer) was cultured in F-12 with 10% FBS. SUM159 (human claudin-low breast cancer) was cultured in F-12 with 5% FBS and supplements (5 μg/mL insulin and 1 μg/mL hydrocortisone). MDA-MB-453 (human HER2-enriched breast cancer) was cultured in DMEM with 10% FBS. SKBR3 (human HER2-enriched breast cancer) was cultured in RPMI with 10% FBS and 1% sodium pyruvate. p53^−^ T11 (murine claudin-low breast cancer) was maintained in RPMI 1640 medium with 10% FBS. BRCA^−^ B.15 (murine basal-like breast cancer) was maintained in RPMI 1640 medium with 10% FBS.

### Cell-viability assays

Cell viability was determined by the Luminescent Cell Viability Assay according to the supplier’s protocol. Cells were plated in 96-well culture plates and incubated at 37 °C and 5% CO_2_ for 24 h. For the dose–response assays, media was discarded and replaced with media containing indicated concentrations of bee venom or peptide and cultured for 24 h. For the cell viability over 60 min, cells were treated with the IC_50_ of honeybee venom or melittin for each cell line for short time intervals over 1 h, and the viability determined immediately after treatment. To determine viability, cells were incubated with CellTiter-Glo (CTG) 2.0 Reagent for 10 min. Cell viability was quantified by measuring luminescence using an EnVision 2102 Multilabel Reader (PerkinElmer). Experiments were conducted in biological replicates (*n* = 3).

### Production of a primary monoclonal antibody against melittin

Antibody production was performed in accordance with protocols approved by the Animal Ethics Committee of the Harry Perkins Institute of Medical Research. Female A/J mice were immunized with honeybee venom collected in Australia. Mice received intraperitoneal injections of 12 μg of venom in Complete Freund’s Adjuvant (Difco), followed by a boost in Incomplete Freund’s Adjuvant on Day 29 and an aqueous boost in PBS at 7 μg/mouse on Day 49. Mice were bled at Day 60, and sera were tested by ELISA. The best responder was boosted with 7 μg of honeybee venom in PBS 4 days prior to fusion. Spleen cells were fused with Sp2/O myeloma cells according to standard procedures^82^. Antibody-containing supernatants were screened by ELISA. Hybridoma clone 3B9 was selected for further study. The antibody was produced by growing the hybridoma cells in bioreactors in Hybridoma Serum Free Medium (Gibco). The antibody was purified by protein G-Sepharose chromatography. Purified antibody was dialyzed in PBS (pH 7.3). The antibody was henceforth referred to as the anti-melittin antibody (3B9).

### Enzyme-linked immunosorbent assay (ELISA)

Venoms and peptides were plated out into clear curve-based 96-well plates at 5 μg/mL in carbonate buffer and incubated at 4 °C for 24 h. The liquid was removed, and the plates washed three times in a solution of 0.05% TWEEN-20 (“Tween-20,” Sigma-Aldrich) in PBS. The primary antibodies were added to the wells with 1:2 dilutions starting from 10 μg/mL in diluent (0.1% bovine serum albumin (BSA) in PBS), and incubated for 1 h at room temperature. The primary antibodies were removed, and the plates washed three times in 0.05% Tween-20 in PBS. The polyclonal goat anti-mouse IgG γ-chain-specific secondary antibody was added to the wells (1:1000 in diluent) and incubated for 1 h at room temperature. The primary antibodies were removed, and the plates washed three times in 0.05% Tween-20 in PBS. ELISA-developing buffer, a solution of purified water containing 10% citric acid (pH 4.2), 2% ABTS, and 0.1% H_2_O_2_, was added to the wells, and plates were incubated in the dark at room temperature for 15 min. Absorbance was recorded at 405 nm using the VICTOR Light plate reader with Wallac 1420 Manager Software (PerkinElmer). The control was the mouse monoclonal IgG antibody (28/00 8C1-6) that reacts with human IL-12, applied to the melittin peptide on the ELISA plate. Experiments were conducted in biological replicates (*n* = 3).

### Anti-melittin antibody competition experiments

HDFa and SUM159 cells were plated in 96-well culture plates and incubated at 37 °C and 5% CO_2_ for 24 h. Increasing concentrations of the anti-melittin antibody were incubated with the IC_50_ concentrations of honeybee venom or melittin for each cell line for 1 h at room temperature, and then added to the cells for 24 h. Cell viability was determined as described in “Cell viability assays”. Experiments were conducted in biological replicates (*n* = 3).

### Western blot

Cells were plated onto 6-well plates at a density of 300,000 cells/well and incubated at 37 °C and 5% CO_2_ for 24 h. Cell culture experiments were conducted as described, and then the standard Western blot protocol was followed as described herein. Cells were washed with cold PBS and lysed with cold protein lysis buffer (2% sodium dodecyl sulfate (SDS), 125 mmol/L Tris-HCl, pH 6.8). Samples were sonicated for 10 s at 10 mA, and protein concentrations quantified with the Detergent Compatible Protein Assay (Bio-Rad). Equal amounts of proteins were mixed with loading buffer (Laemmli Sample Buffer, Bio-Rad) supplemented with the reducing agent dithiothreitol (DTT). Protein samples were denatured by boiling at 95 °C for 5 min, loaded into Mini-PROTEAN precast gels (Bio-Rad) and subjected to electrophoresis at 100 V, and then transferred to PVDF membranes (Bio-Rad) with the Trans-Blot Turbo Transfer System (Bio-Rad) for 7 min. Membranes were incubated with TBST (20 mM Tris-HCl, pH 7.4, 150 mM NaCl, and 0.1% Tween-20) with 5% nonfat milk to block nonspecific binding. Membranes were incubated overnight at 4 °C with the primary antibodies diluted in 3% BSA and 0.02% sodium azide. The signal was detected with Luminata Crescendo Western HRP Substrate (Millipore) with the ChemiDoc MP Imaging System (Bio-Rad) running Image Lab Software (Bio-Rad, Version 6). Western blots were derived from the same experiment and processed in parallel. Uncropped scans of the Western blots are provided in Supplementary Figs. 10–15.

### Flow cytometry

Apoptosis and necrosis were assessed using the Annexin V-FITC Apoptosis Detection Kit I (BD Biosciences) according to the manufacturer’s protocol. SUM159 cells were plated in 6-well culture plates for 24 h. Media was then discarded and replaced with media containing honeybee venom or melittin (IC_50_ concentrations) and cultured for 60 min. Cells were collected with trypsin and media, and centrifuged (1000*g*, 5 min, 24 °C), washed with cold PBS, centrifuged (1000*g*, 5 min, 24 °C), and resuspended in 1× binding buffer. Cells were prepared to a concentration of 1 million cells/mL in 1× binding buffer. Samples were incubated with FITC and PI (5 µL of each) in the dark for 15 min. The presence of live, dead, apoptotic, or necrotic cells was assessed with the BD Accuri C6 Cytometer (BD Biosciences, San Jose, USA) with BD Accuri C6 software, and analyzed with FlowJo^™^ (Ashland, USA, Windows Version 7). Experiments were conducted in biological replicates (*n* = 3). The gating strategies are presented in Supplementary Fig. 16.

### Live-cell microscopy

SKBR3 cells were plated into a glass-bottom microwell dish (10 × 35 mm, MatTek) and incubated for 24 h. The microwell dish was left to equilibrate in a NIKON Eclipse Ti confocal microscope stage-top incubation chamber (37 °C and 5% CO_2_) for 20 min. The 20× objective was used with Kohler alignment, and images were taken every minute from 10 min before to 1 h after treatment with the IC_50_ of honeybee venom collected in Australia. The authors acknowledge the facilities and scientific and technical assistance offered by the National Imaging Facility, a National Collaborative Research Infrastructure Strategy (NCRIS) capability, as well as the Australian Microscopy & Microanalysis Research Facility, both at the Centre for Microscopy, Characterization and Analysis (CMCA), UWA, a facility funded by the University, State and Commonwealth Governments.

### Scanning electron microscopy

Glass coverslips (12-mm diameter, Menzel, Thermo Fisher Scientific) were coated with poly-l-lysine hydrobromide (Sigma-Aldrich) for 20 min and then washed twice with purified water. SUM159 cells were plated onto the glass slides at a density of 62,500 cells/well and incubated at 37 °C and 5% CO_2_ for 24 h. Cells were washed twice with PBS and then treated with vehicle or the IC_50_ concentrations of honeybee venom and melittin for 1 h. Cells were washed twice with PBS, then fixed with 4% formaldehyde in PBS for 25 min, and then washed again three times with PBS. In preparation for microscopy, the samples were immersed in 2.5% glutaraldehyde and were incubated at 4 °C for 2 h. The samples were washed with deionized water and immersed in increasing concentrations of ethanol (50%, 70%, 95%, 100%, and then 100% absolute “dry” ethanol). Between each immersion, the samples were dehydrated in a specialized microwave (PELCO, BioWave 34700 Laboratory Microwave System). The dehydration process was completed with a Critical Point Drying Apparatus E3000 to replace the ethanol in the sample with supercritical CO_2_. The processed coverslips were mounted on SEM mounts (ProSciTech) with carbon tabs. The samples were coated with 3-nm platinum to make them electronically conductive before being visualized under the scanning electron microscope (Zeiss 1555 VP-FESEM) at CMCA, UWA. Images were taken with the in-lens detector at 2.6-mm working distance, 30-μm aperture, and an accelerating voltage of 5 kV. Images were analyzed with the image analysis software FIJI (ImageJ)^83^.

### Immunofluorescence

Glass coverslips (12-mm diameter, Menzel, Thermo Fisher Scientific) were placed in 24-well plates and coated with poly-l-lysine (Sigma-Aldrich) for 20 min and then washed twice with purified water. SUM159 cells were plated onto the glass slides and incubated at 37 °C and 5% CO_2_ for 24 h. Cells were treated for 30 min with vehicle, or the IC_50_ of honeybee venom, melittin, RGD1-melittin, and the equivalent molar concentration as melittin for DEDE-melittin. Cells were washed twice with PBS, then fixed with 4% paraformaldehyde in PBS for 25 min, and then washed again three times with PBS. Nonspecific antibody binding was blocked using 5% Normal Goat Serum (Thermo Fisher Scientific) in PBS for 1 h at room temperature. Primary antibodies were added to the cells, including the monoclonal anti-melittin antibody (5 μg/mL) and 1:500 of anti-EGFR [EP38Y] (Abcam). The samples were incubated with gentle rocking at 4 °C overnight. The cells were washed three times with PBS, and then incubated with 1:500 of Alexa Fluor 488 goat anti-mouse secondary antibody, 1:500 of Alexa Fluor 594 goat anti-rabbit secondary antibody, and Hoechst (1:5000) in PBS at room temperature for 1 h. The samples were washed three times with PBS and mounted onto glass coverslips with SlowFade Diamond Antifade Mountant (Thermo Fisher Scientific). Slides were imaged using the confocal fluorescence Nikon Ti-E inverted microscope. Images were taken using a 20× air objective (NA 0.75), and sequential excitation using wavelengths of 405 nm (Hoechst 34580), 488 nm (Alexa Fluor 488 secondary antibody), and 561 nm (Alexa Fluor 594 secondary antibody). Images were collected using NIS-C Elements Software and processed using FIJI (ImageJ) at CMCA^83^.

### Bioluminescence resonance energy transfer (BRET)

Receptor–ligand interactions were assessed with BRET, using a method similar to that described previously^84,85^. BRET involves the nonradiative transfer of energy (dipole–dipole) between two proteins or molecules of interest labeled with either a donor luciferase or an acceptor fluorophore after substrate oxidation by the luciferase and subsequent emission of light^54^. FITC tags were conjugated to the N terminus of melittin (FITC-melittin) and DEDE-melittin (FITC–DEDE-melittin). HEK293 cells stably expressing the SV40 large T antigen (HEK293FT) were plated onto 6-well plates at a density of 550,000 cells/well for 24 h. HEK293FT cells were transfected with plasmids containing cDNA for NanoLuc-EGFR using FuGENE. Briefly, plasmid cDNA was incubated for 10 min at room temperature with a mix of transfection reagent and serum-free DMEM at a ratio of 10 ng/μL NanoLuc-EGFR: 4 μL of FuGENE: 100 μL of SFM. The mix was added to the HEK293FT cells at a final concentration of 10 ng/μL NanoLuc-EGFR per well of the 6-well plate, and cells incubated for 24 h. Cells were washed with PBS and detached with trypsin, then collected in media containing 5% fetal calf serum in phenol red-free DMEM. Cells were plated at 50,000 cells/well into poly-l-lysine-coated 96-well white plates and incubated for 24 h. For both saturation and kinetic BRET assays, two filters were used to simultaneously measure the short- and long-wavelength luminescence corresponding to the emission wavelengths of the donor and acceptor molecules, respectively.

For real-time ligand association kinetics experiments, the media was removed from the cells, which were then incubated with 50 μL/well of the NanoLuc substrate furimazine to a final concentration of 10 μM diluted in Hank’s Balanced Salt Solution (HBSS). The cells were then equilibrated in the CLARIOstar plate reader (BMG Labtech, Australia) for 5 min to record basal readings. The ligands (TAMRA-EGF, FITC-melittin, and FITC–DEDE-melittin) were then added to a range of correct final concentrations, and NanoBRET recordings taken every 90 s for 60 min at 37 °C. For the saturation experiments, the media was removed from the cells, and a range of concentrations of TAMRA-EGF, FITC-melittin, and FITC–DEDE-melittin added in the presence or absence of a competing concentration (1 µM) of unlabeled EGF and incubated at 37 °C for 60 min in the dark. Furimazine was added at a final concentration of 10 μM. Recordings were made using the LUMIstar Omega (BMG Labtech, Australia). Data are presented as the “raw BRET ratio,” derived from the ratio of the long-wavelength emission (acceptor) over the short-wavelength emission (donor). Experiments were conducted in biological replicates (*n* = 3).

### Analysis of combined drug effects

Honeybee venom or melittin was combined with docetaxel and administered at the concentrations indicated in a nonconstant ratio in T11 cells for 24 h. Cell viability was assessed using CellTiter-Glo as mentioned previously. The combined effect of honeybee venom or melittin with docetaxel was assessed by the median dose-effect method using the CompuSyn Software (ComboSyn). This method determines a CI based on the effect of a combination between two agents (where CI < 1 is synergistic, CI > 1 is antagonistic, and CI = 1 is additive)^56^. Experiments were conducted in biological replicates (*n* = 3).

### Animal model and treatments

These animal experiments were performed in accordance with protocols approved by the Animal Ethics Committee of UWA. To simulate an advanced model of claudin-low breast cancer, 2.5 × 10^5^ T11 cells were suspended in serum-free media and BD Matrigel Matrix High Concentration (BD Bioscience) in a 1:1 ratio to a total volume of 100 μL and injected subcutaneously into the flanks of 5-week-old BALB/cJ females (Animal Resources Centre, WA, Australia) using a 26-G needle. The T11 cells used were lentivirally transduced with the ZsGreen-luciferase construct and sorted three times to achieve an enrichment superior than 99% of luciferase-positive cells. Melittin was suspended in Milli-Q water + 5% dextrose. Docetaxel (in powder) was suspended in 25% TWEEN 80 (Sigma-Aldrich), and 75% of a mixture of 15.25:84.75 (v/v) solution of absolute ethanol and purified water and kept at −20 °C. Immediately before the treatments, docetaxel was freshly diluted in Milli-Q water + 5% dextrose at the required final concentration. Three days after the generation of T11 tumors (~50 mm^3^), mice were randomized into 4 groups (*n* = 12 mice/group). The treatments were injected intratumorally on days 3, 5, 7, 9, 11, 13, and 15 post inoculation of T11 cells, with vehicle, melittin (5 mg/kg), docetaxel (7 mg/kg), or a combination of melittin (5 mg/kg) and docetaxel (7 mg/kg). Animals were monitored for tumor size every 2 days, and volumes calculated by the modified ellipsoid formula (volume = width^2^ × length/2). Animals were humanely sacrificed when the tumors reached 800 mm^3^.

### Immunohistochemical analysis of the tumors

Tumor tissues were fixed in 4% paraformaldehyde, washed three times in PBS, and left in 70% ethanol. Tumors were embedded in paraffin, and 5-μm sections were prepared. For hematoxylin/eosin staining, slides were dewaxed, hydrated using a decreasing solution bank of ethanol, stained with Gill’s hematoxylin, dehydrated using 70% ethanol, stained with eosin, further dehydrated using 100% ethanol, cleared using toluene, and mounted in coverslips using Acrymount IHC mounting media (StatLab). Tumor cell apoptosis was determined in tissue sections by TUNEL assay (In Situ Cell Death Detection Kit, Roche).

### Bioluminescence imaging

To accurately track changes in in vivo tumor growth with the treatments, we performed bioluminescence analysis using the Caliper IVIS Lumina II imaging system at CMCA, UWA. The analyses were conducted every 2 days after the generation of tumors. Mice were injected intraperitoneally with 200 µL of D-Luciferin (Cayman Chemical) at the final concentration of 150 mg/kg dissolved in PBS prior to being anesthetized at 4% isoflurane. Once anesthetized, mice were placed inside the prewarmed chamber of the bioluminescence imager and imaged 7–12 min after injection, under 2% isoflurane, until bioluminescence signal intensity had reached a steady state.

### Statistical analysis

All data were derived from multiple experiments conducted at least in triplicate. Statistical analyses were performed with GraphPad Prism v8 (GraphPad Software Inc.), Office Excel 365 (Microsoft), and SPSS Predictive Analytics Software (IBM, Version 26). For the cell-viability assays, data were normalized to the average luminescence of the vehicle condition, which was considered 100% viability, with the IC_50_s derived in GraphPad Prism. For the immunohistochemistry in the treated T11 tumors to detect p-HER2 (Tyr1248) and p-EGFR (Tyr1068), vehicle was normalized to 100%. Where appropriate and as indicted in the main text, statistical significance was determined using unpaired two-tailed Student’s t tests, unpaired one-way ANOVA with Tukey’s HSD post hoc test correcting for multiple comparisons, two-way ANOVA with repeated measures followed by Sidak’s or Tukey’s multiple-comparison test, or a generalized linear model (GLM). For all tests, differences were considered significant at *p* < 0.05 (*), *p* < 0.01 (**), and *p* < 0.001 (***).

### Reporting summary

Further information on research design is available in the Nature Research Reporting Summary linked to this article.

## Supplementary information

Supplementary Figures

Reporting Summary

## Data Availability

All data generated or analyzed during this study are included in this published article (and its supplementary information files). The anti-melittin antibody developed at the Monoclonal Antibody Facility at the Harry Perkins Institute of Medical Research could be made available once appropriate agreements are in place.
