# Supplementary material for: Honeybee venom and melittin suppress growth factor receptor activation in HER2-enriched and triple-negative breast cancer
Source: NPJ Precis Oncol. 2020 Sep 1;4:24. doi: 10.1038/s41698-020-00129-0 (PMC7463160; doi:10.1038/s41698-020-00129-0)
Supplement: Supplementary file 1 — Supplementary Figures [file 41698_2020_129_MOESM1_ESM.pdf]

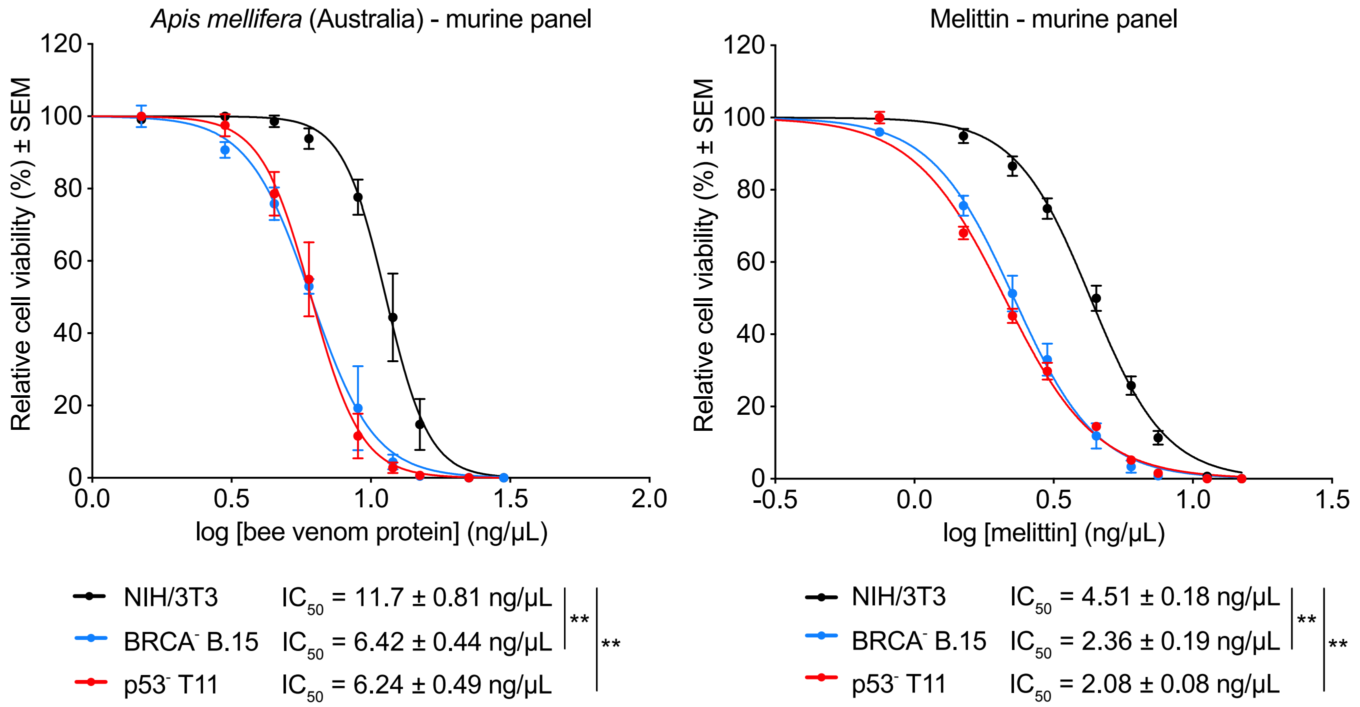

Supplementary Figure 1. Related to Figure 1. Honeybee venom and melittin potently reduce viability in murine TNBC cells. Cell viability assays of a panel of murine non-transformed (NIH/3T3), basal-like breast cancer (BRCA$^-$ B.15), and claudin-low breast cancer (p53$^-$ T11) cell lines treated with honeybee venom collected in Australia (left) or melittin (right). Data are represented as mean ± SEM (*n*=3). Differences were considered significant at $p<0.05$ (*), $p<0.01$ (**) and $p<0.001$ (***) (one-way ANOVAs).

Quantification of the Western blot in Figure 2a

	Cleaved caspase-3	Tubulin		
**Condition**	**Total Band Volume (Intensity)**	**Total Band Volume (Intensity)**	**Ratio Cleaved caspase-3:Tubulin**	**Normalised ratio**
Untreated	155474	9047195	0.02	0.02
Honeybee venom 18 hours	1878180	8906496	0.21	0.25
Honeybee venom 24 hours	2308768	6923819	0.33	0.40
Melittin 18 hours	5724780	6738875	0.85	1.01
Melittin 24 hours	5901532	7050516	0.84	1.00

Supplementary Figure 2. Related to Figure 2. Normalized expression of cleaved caspase-3 (CL-csp-3) across samples relative to α-Tubulin expression in SUM159 cells. A comparison analysis of the relative abundance of CL-csp-3 across the lanes of the Western blot presented in Figure 2a, quantified with Image Lab software.

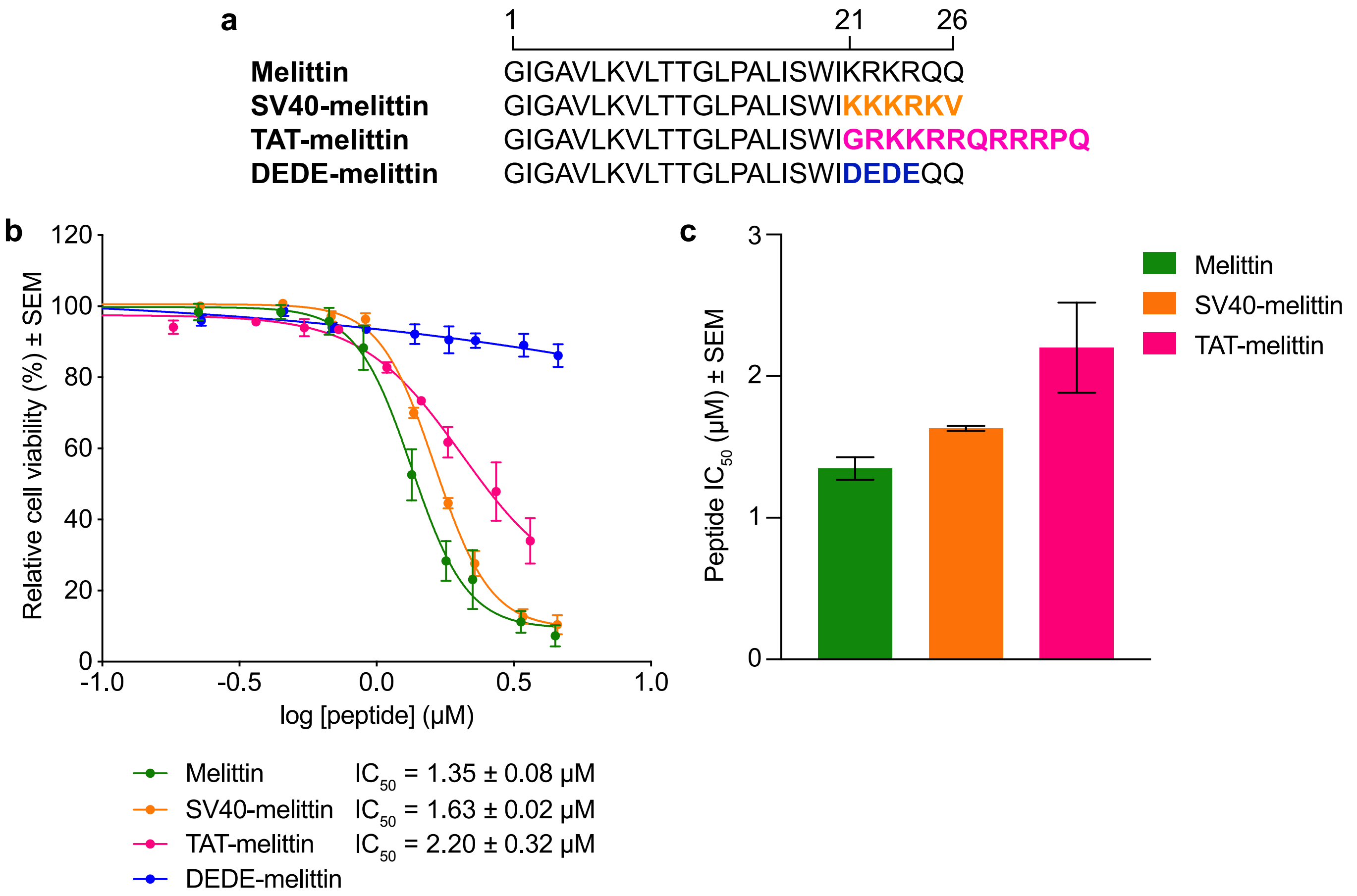

Supplementary Figure 3. Related to Figure 3. The positively charged residues residing in the C-terminal α-helix of melittin mediate anti-cancer activity. (**a**) The amino acid sequence of melittin, SV40-melittin, TAT-melittin and DEDE-melittin. (**b**) Cell viability assays of T11 cells treated with melittin, SV40-melittin, TAT-melittin and DEDE-melittin for 24 hours. (**c**) The IC_{50} values of melittin, SV40-melittin and TAT-melittin. Data are represented as mean ± SEM.

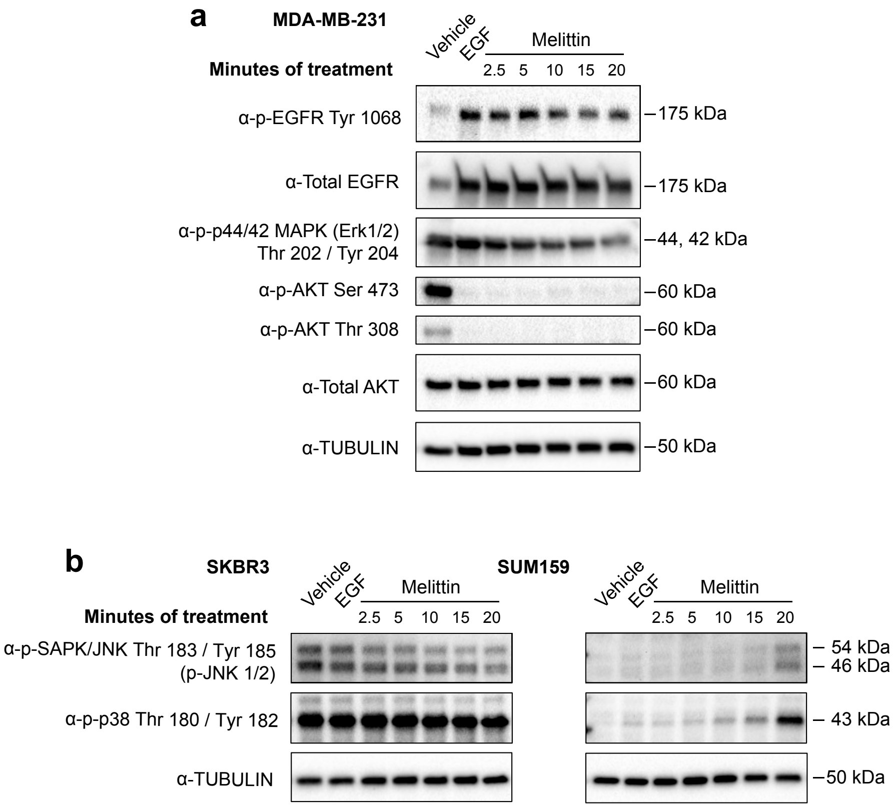

Supplementary Figure 4. Related to Figure 4. Melittin suppresses RTK phosphorylation and inhibits survival pathways in TNBC and HER2-enriched cells. (**a**) Phosphorylation kinetics of EGFR, MAPK, and Akt pathways after treatment with melittin in MDA-MB-231 cells and (**b**) downstream members of the MAPK family (JNK1/2 and p-p38 MAPK) in SKBR3 and SUM159 cells, assessed by immunoblotting.

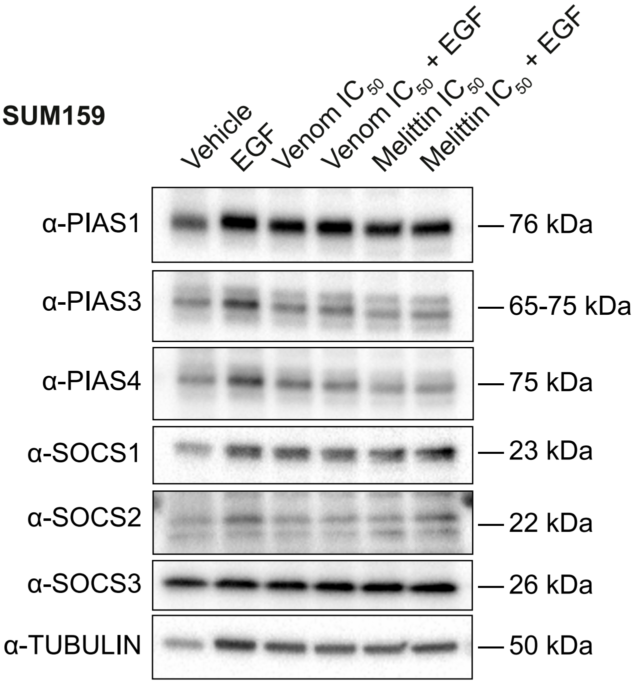

Supplementary Figure 5. Related to Figure 4. Honeybee venom and melittin did not modulate the levels of JAK/STAT inhibitors in TNBC cells. Western blot for the detection of JAK/STAT pathway inhibitors in SUM159 cells treated with vehicle, honeybee venom or melittin for 60 minutes, with and without 20 ng/mL EGF for 5 minutes.

**Peptide**	**Amino acid sequence**
FITC-EN1-mutant	FITC-PLVAPAAVYCTRYSDR

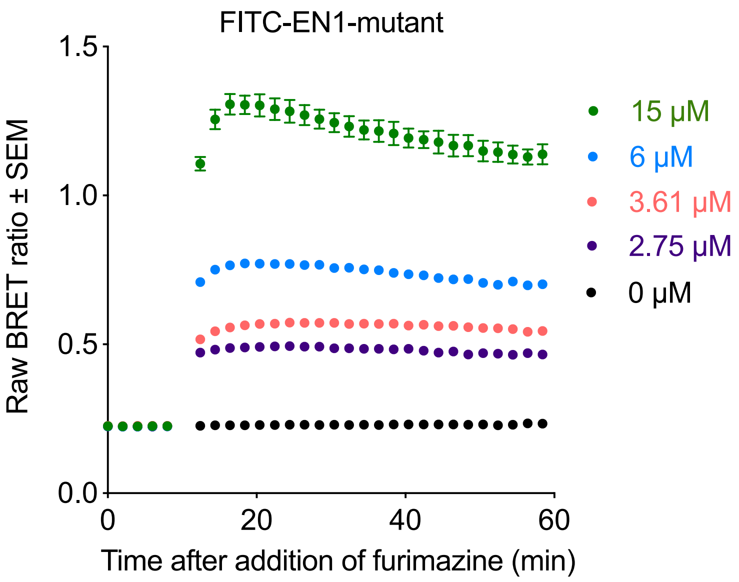

Supplementary Figure 6. Related to Figure 4. A non-specific peptide targeting Engrailed 1 (EN1) exhibited similar BRET kinetics to FITC-DEDE-melittin. Kinetic analysis of FITC-EN1-mutant interaction with NanoLuc-EGFR by bioluminescence resonance energy transfer (BRET) in HEK293FT cells. FITC-EN1-mutant was added after the cells were equilibrated in the reader with the NanoLuc-substrate furimazine for 5 minutes. EN1 refers to an interference peptide targeting the human Engrailed 1 transcription factor.

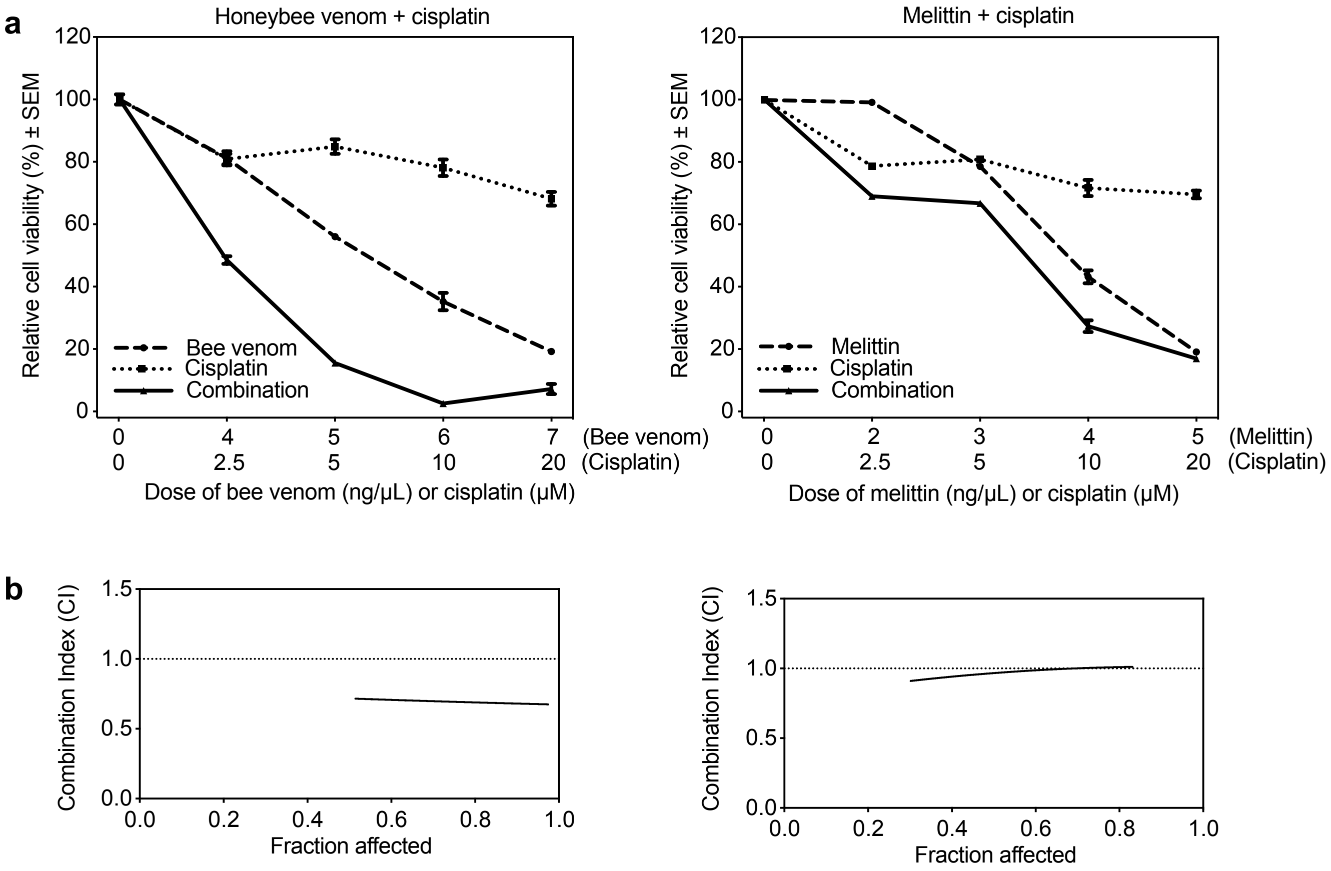

Supplementary Figure 7. Related to Figure 5. Honeybee venom and melittin were additive and/or synergistic with cisplatin in reducing T11 cell viability. (**a**) Cell viability assays of breast cancer cells (T11) treated with honeybee venom and melittin alone and in combination with cisplatin for 24 hours. Representative plots of the combination treatments are represented as mean ± SEM (*n*=3). (**b**) Combination Index graphs obtained for different fractions of cells affected in each combination, calculated using CompuSyn software. A synergistic drug combination is indicated by CI<1.

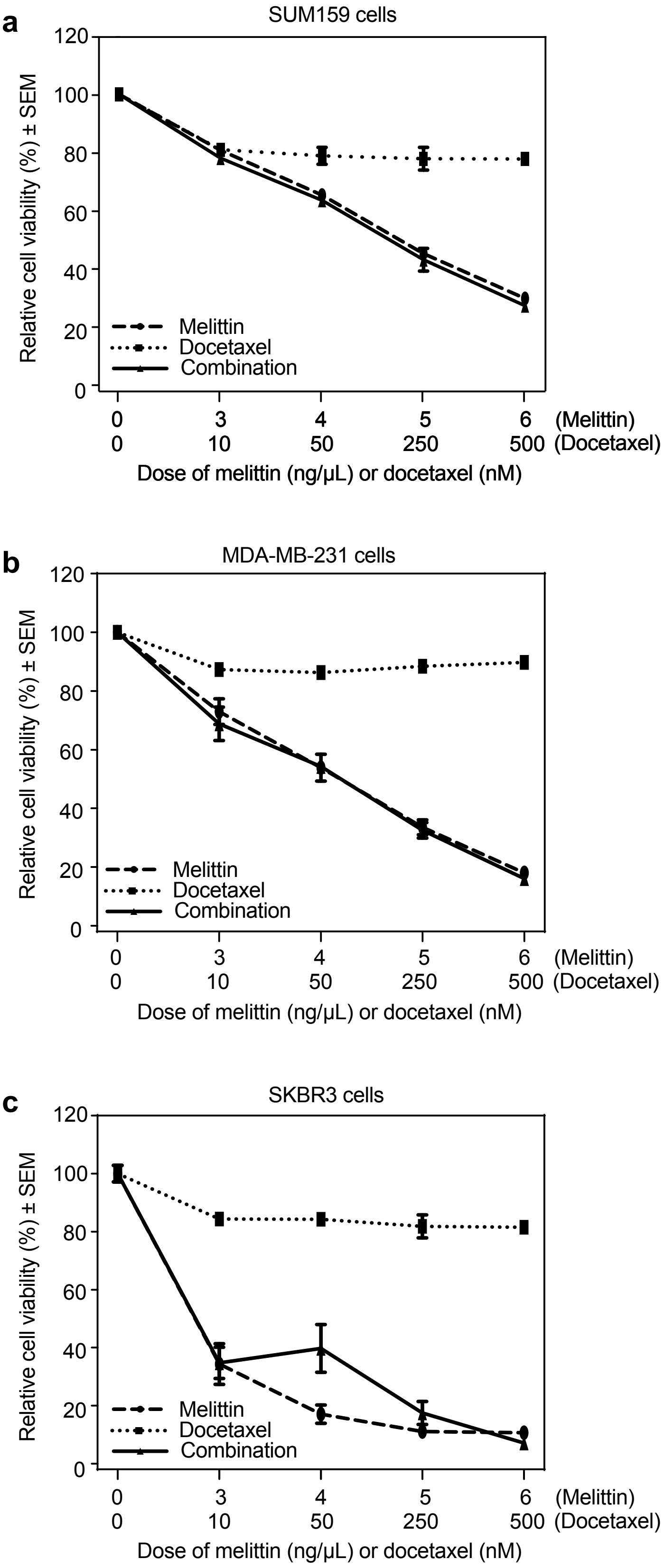

Supplementary Figure 8. Related to Figure 5. Melittin and docetaxel combination treatments in SUM159, MDA-MB-231, and SKBR3 breast cancer cells. Cell viability assays of (**a**) SUM159, (**b**) MDA-MB-231, and (**c**) SKBR3 cells treated with melittin alone, docetaxel alone, and with the combination treatment of melittin and docetaxel for 24 hours. Representative plots of the combination treatments are presented as mean ± SEM (*n*=3).

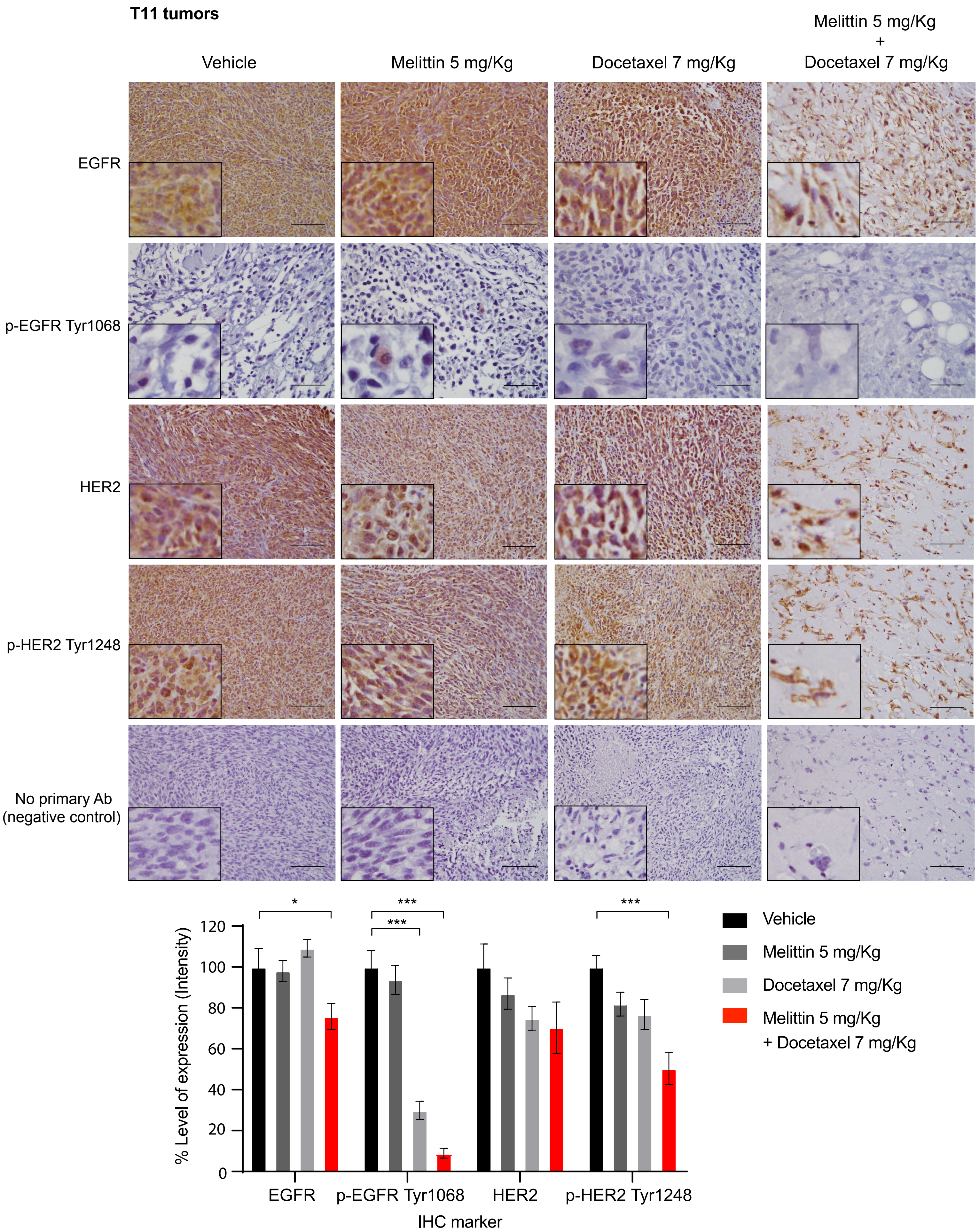

Supplementary Figure 9. Related to Figure 5. Transmodulation of p-HER2 (Tyr1248) and p-EGFR (Tyr1068) *in vivo*. Representative images and quantification of immunohistochemistry in tumor biopsies from mice extracted on day 14 post-T11 cells inoculation, evaluating the effect of single or combined inhibition with melittin and docetaxel on the expression levels of EGFR and HER2 and reduction of p-EGFR and p-HER2. Scale bars represent 100 µm. Data are represented as mean ± SEM. Differences were considered significant at $p<0.05$ (*), $p<0.01$ (**) and $p<0.001$ (***) (one-way ANOVAs).

a SUM159 (18 - 24 hours)

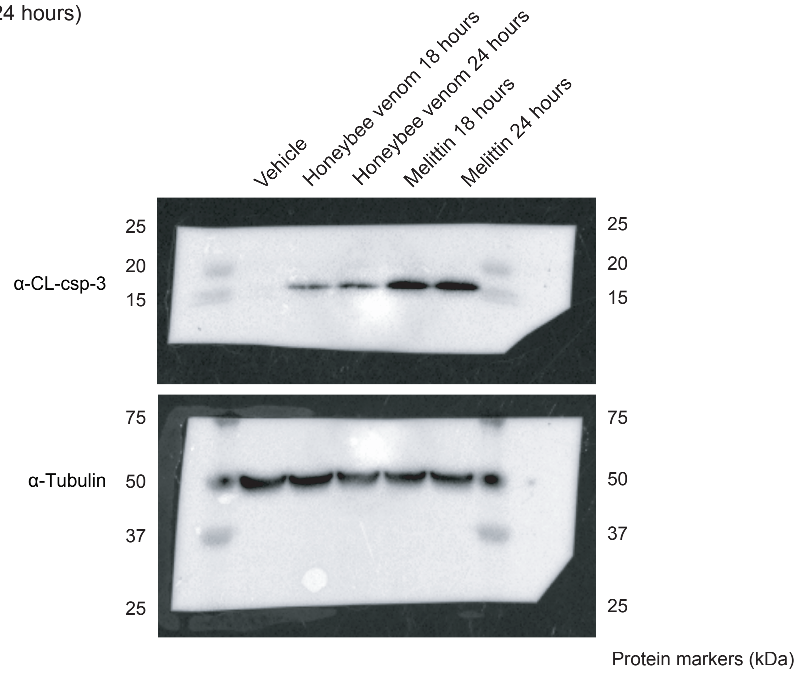

b SUM159 (24 hours)

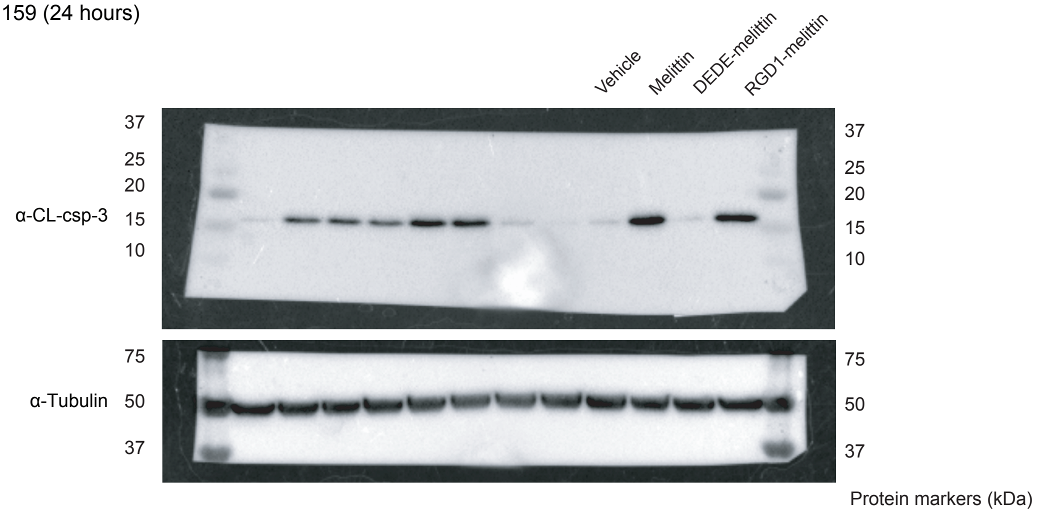

Supplementary Figure 10. Related to Figures 2 and 3. Uncropped scans of the CL-csp-3 Western blots. Uncropped scans of the Western blots of CL-csp-3 and α-Tubulin in SUM159 cells presented in (**a**) Figure 2a and (**b**) Figure 3d.

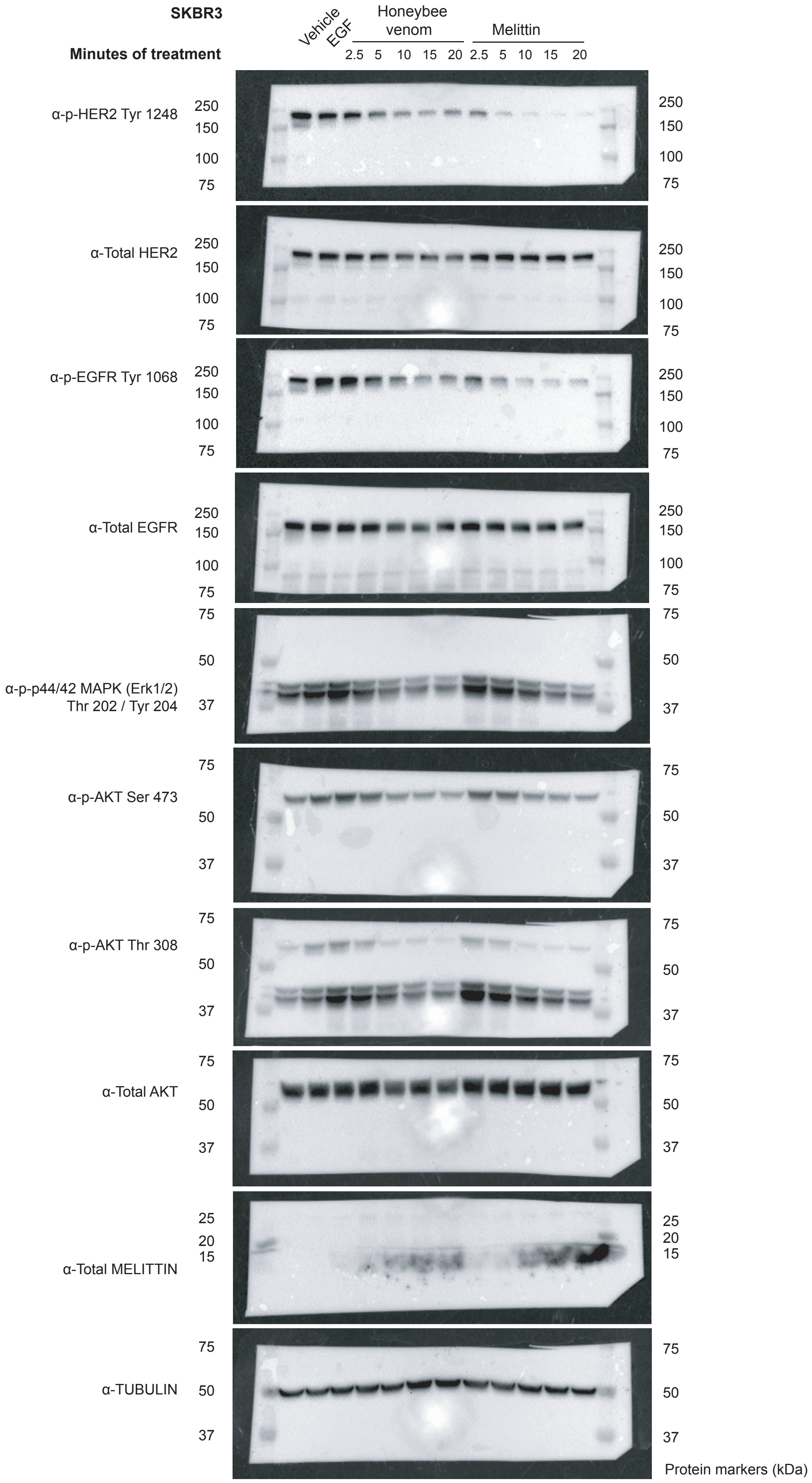

Supplementary Figure 11. Related to Figure 4. Uncropped scans of the pathway Western blots. Uncropped scans of the Western blots of EGFR and HER2 pathway analysis in SKBR3 cells presented in Figure 4a, left.

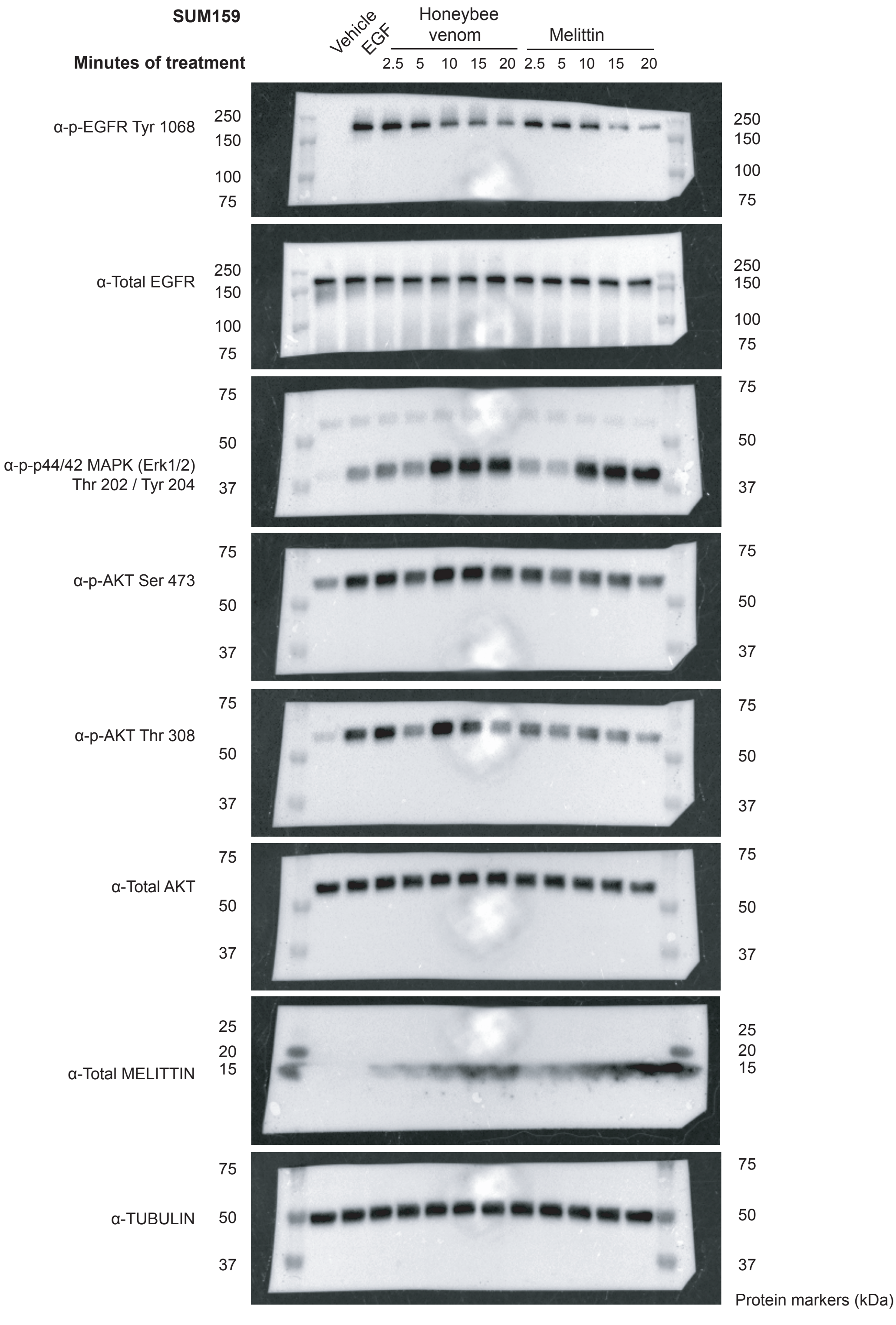

Supplementary Figure 12. Related to Figure 4. Uncropped scans of the pathway Western blots. Uncropped scans of the Western blots of EGFR pathway analysis in SUM159 cells presented in Figure 4a, right.

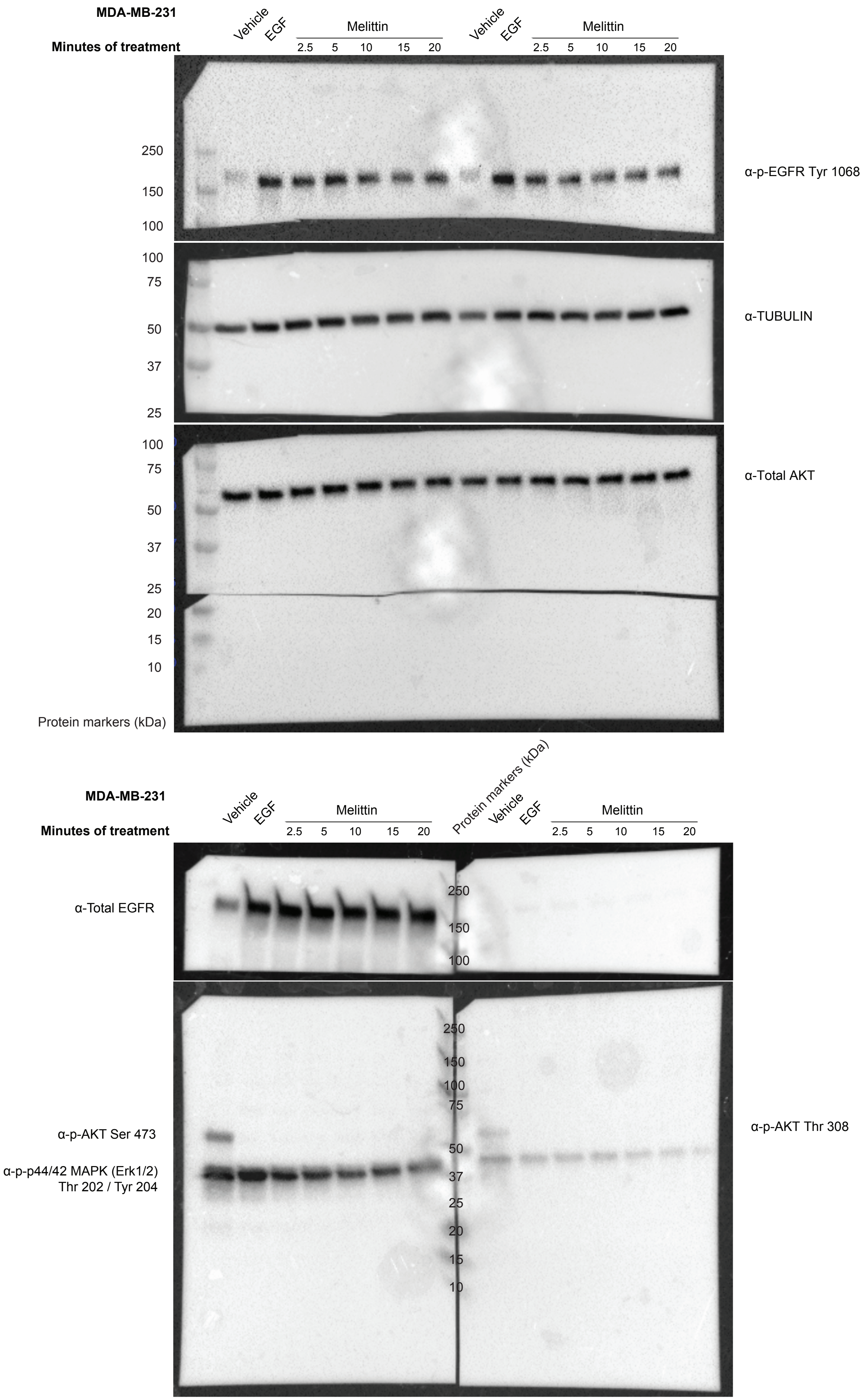

Supplementary Figure 13. Related to Supplementary Figure 4. Uncropped scans of the pathway Western blots. Uncropped scans of the Western blots of EGFR pathway analysis in MDA-MB-231 cells presented in Supplementary Figure 4a.

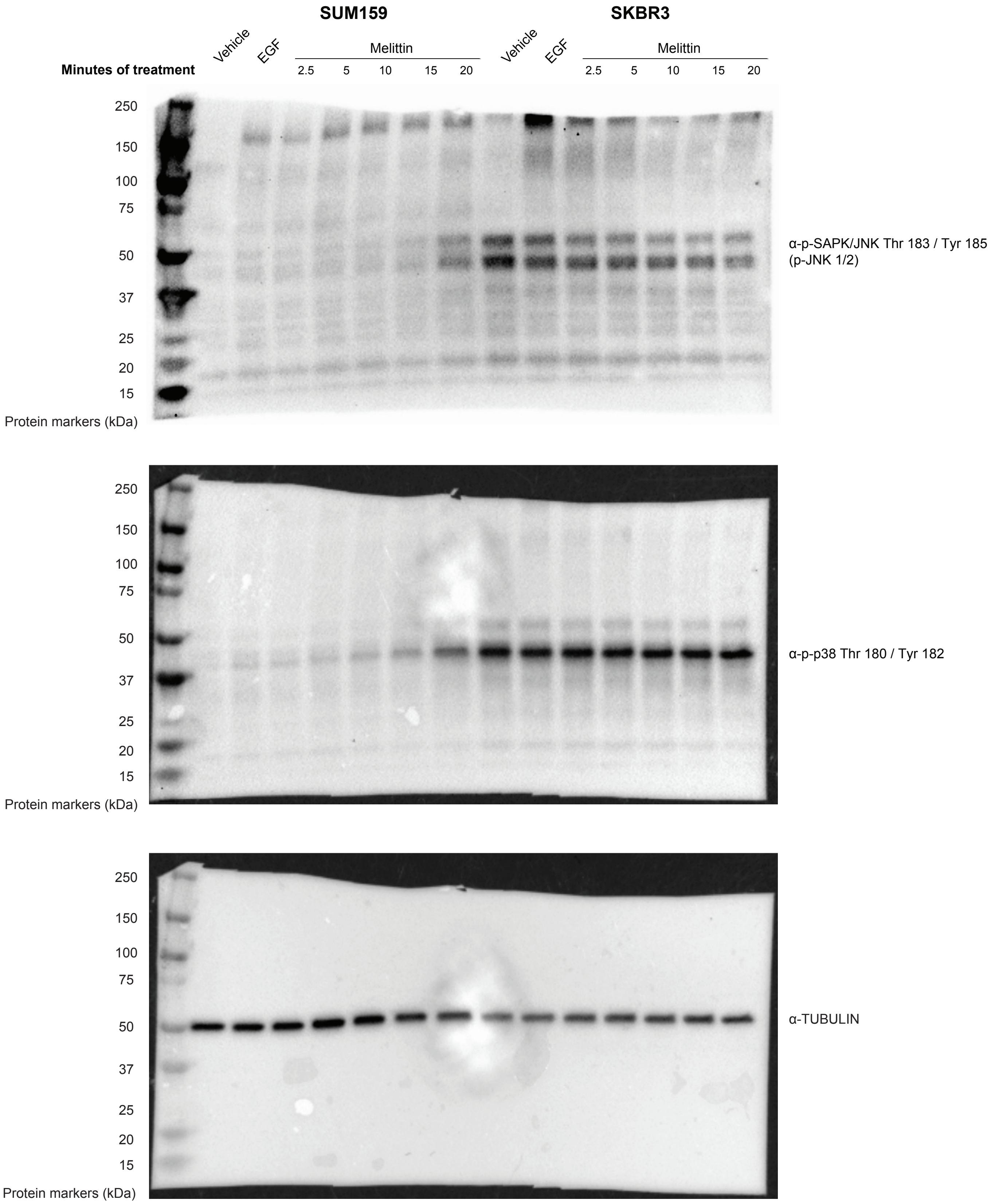

Supplementary Figure 14. Related to Supplementary Figure 4. Uncropped scans of the pathway Western blots. Uncropped scans of the Western blots of p-SAPK/JNK and p-p38 pathway analysis in SUM159 and SKBR3 cells presented in Supplementary Figure 4b.

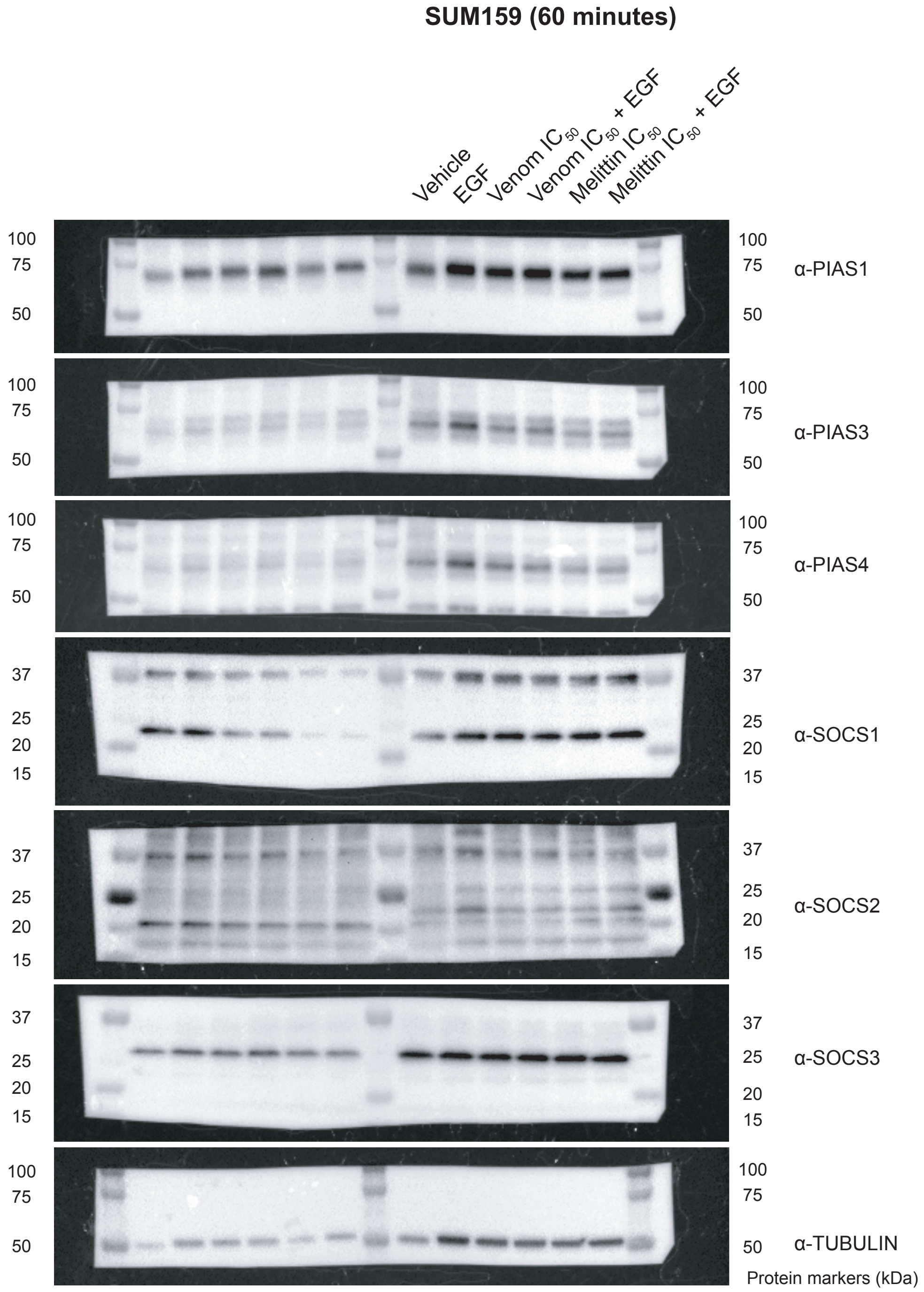

Supplementary Figure 15. Related to Supplementary Figure 5. Uncropped scans of the JAK/STAT pathway Western blots. Uncropped scans of the Western blots of JAK/STAT pathway inhibitors in SUM159 cells presented in Supplementary Figure 5.

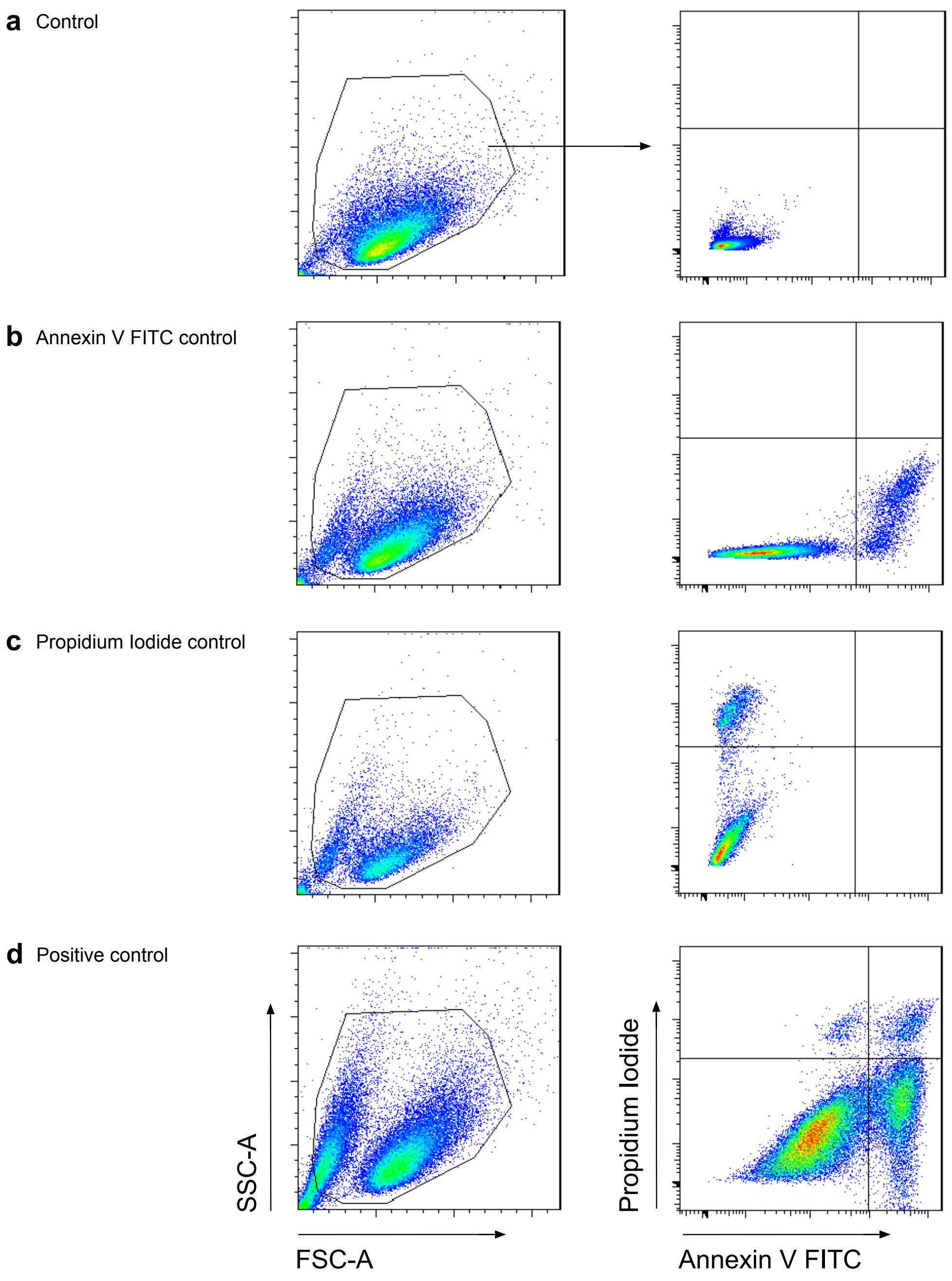

Supplementary Figure 16. Related to Figure 2. Gating strategies used for flow cytometry analysis according to the supplier's protocol (BD Biosciences). Gating strategy for *in vitro* cultures of SUM159 TNBC cells after a 60-minute treatment with (**a**) control, (**b**) Annexin V FITC control, (**c**) Propidium Iodide control, and (**d**) Positive control, presented in Figure 2b.
