# Supplementary material for: Honeybee venom and melittin suppress growth factor receptor activation in HER2-enriched and triple-negative breast cancer
Source: NPJ Precis Oncol. 2020 Sep 1;4:24. doi: 10.1038/s41698-020-00129-0 (PMC7463160; doi:10.1038/s41698-020-00129-0)
Supplement: Supplementary file 2 — Reporting Summary [file 41698_2020_129_MOESM2_ESM.pdf]

nature research

Corresponding author(s): Pilar Blancafort

Last updated by author(s): 11 July, 2020

Reporting Summary

Nature Research wishes to improve the reproducibility of the work that we publish. This form provides structure for consistency and transparency in reporting. For further information on Nature Research policies, see our Editorial Policies and the Editorial Policy Checklist.

Statistics

For all statistical analyses, confirm that the following items are present in the figure legend, table legend, main text, or Methods section.

n/a	Confirmed	
☐	☒	The exact sample size (*n*) for each experimental group/condition, given as a discrete number and unit of measurement
☐	☒	A statement on whether measurements were taken from distinct samples or whether the same sample was measured repeatedly
☐	☒	The statistical test(s) used AND whether they are one- or two-sided<br>*Only common tests should be described solely by name; describe more complex techniques in the Methods section.*
☒	☐	A description of all covariates tested
☒	☐	A description of any assumptions or corrections, such as tests of normality and adjustment for multiple comparisons
☐	☒	A full description of the statistical parameters including central tendency (e.g. means) or other basic estimates (e.g. regression coefficient) AND variation (e.g. standard deviation) or associated estimates of uncertainty (e.g. confidence intervals)
☐	☒	For null hypothesis testing, the test statistic (e.g. *F*, *t*, *r*) with confidence intervals, effect sizes, degrees of freedom and *P* value noted<br>*Give P values as exact values whenever suitable.*
☒	☐	For Bayesian analysis, information on the choice of priors and Markov chain Monte Carlo settings
☒	☐	For hierarchical and complex designs, identification of the appropriate level for tests and full reporting of outcomes
☒	☐	Estimates of effect sizes (e.g. Cohen's *d*, Pearson's *r*), indicating how they were calculated

Our web collection on statistics for biologists contains articles on many of the points above.

Software and code

Policy information about availability of computer code

Data collection	EnVision 2102 Multilabel Reader (PerkinElmer), Image Lab 6.0.1, Endnote X9.2, NIS-C Elements, BD AccuriC6, Millennium Science BioTek PowerWave XS2, VICTOR Light plate reader, ChemiDoc MP Imaging System (Bio-Rad), Zeiss 1555 VP-FESEM microscope, CLARIOstar plate reader (BMG Labtech, Australia), LUMIstar Omega (BMG Labtech, Australia), Caliper IVIS Lumina II
Data analysis	GraphPad Prism 8.4.2, Image Lab 6.0.1, CompuSyn, FIJI (ImageJ), FlowJo™ Software Version 7.6.1, Gen 5 1.11 (Version 1.11.5), Wallac 1420 Manager Software (PerkinElmer)

For manuscripts utilizing custom algorithms or software that are central to the research but not yet described in published literature, software must be made available to editors and reviewers. We strongly encourage code deposition in a community repository (e.g. GitHub). See the Nature Research guidelines for submitting code & software for further information.

Data

Policy information about availability of data

All manuscripts must include a data availability statement. This statement should provide the following information, where applicable:

- Accession codes, unique identifiers, or web links for publicly available datasets
- A list of figures that have associated raw data
- A description of any restrictions on data availability

All data generated or analyzed during this study are included in this published article (and its supplementary information files).

Field-specific reporting

Please select the one below that is the best fit for your research. If you are not sure, read the appropriate sections before making your selection.

☒ Life sciences ☐ Behavioural & social sciences ☐ Ecological, evolutionary & environmental sciences

For a reference copy of the document with all sections, see nature.com/documents/nr-reporting-summary-flat.pdf

Life sciences study design

All studies must disclose on these points even when the disclosure is negative.

Sample size	The number of animals per each group was determined based on previous published data from our lab with the same cancer model (n=12 mice per group)
Data exclusions	No data were excluded
Replication	All the experiments have been repeated at least three times and all the attempts at replication were successful
Randomization	Randomization was performed in the animal experiment by randomly allocating mice harbouring tumors in each experimental group
Blinding	The investigators were not blinded to group allocation since the treatments involved complex treatments e.g synergistic assessments. The study was developed instead in different treatment "arms"

Reporting for specific materials, systems and methods

We require information from authors about some types of materials, experimental systems and methods used in many studies. Here, indicate whether each material, system or method listed is relevant to your study. If you are not sure if a list item applies to your research, read the appropriate section before selecting a response.

Materials & experimental systems

n/a	Involved in the study	
☐	☒	Antibodies
☐	☒	Eukaryotic cell lines
☒	☐	Palaeontology and archaeology
☐	☒	Animals and other organisms
☒	☐	Human research participants
☒	☐	Clinical data
☒	☐	Dual use research of concern

Methods

n/a	Involved in the study	
☒	☐	ChIP-seq
☐	☒	Flow cytometry
☒	☐	MRI-based neuroimaging

Antibodies

Antibodies used

phospho-ErbB2 (Tyr1248), Cell Signaling Technology, catalogue number 2247
phospho-EGFR (Tyr1068), Cell Signaling Technology, catalogue number 2234
phospho-EGFR (Tyr1068), Cell Signaling Technology, clone D7A5, catalogue number 3777
phospho-p44/42 MAPK (Erk1/2) (Thr202/Tyr204), Cell Signaling Technology, catalogue number 4370
phospho-Akt (Ser473), Cell Signaling Technology, catalogue number 4060
phospho-Akt (Thr308), Cell Signaling Technology, catalogue number 13038
phospho-SAPK/JNK (Thr183/Tyr185), Cell Signaling Technology, clone 81E11, catalogue number 4668
phospho-p38 MAPK (Thr180/Tyr182), Cell Signaling Technology, clone D3F9, catalogue number 4511
Total AKT, Cell Signaling Technology, catalogue number 9272 and 4685
Cleaved Caspase-3 (Asp175), Cell Signaling Technology, catalogue number 9661
Ki-67, Cell Signaling Technology, catalogue number 9449
α-Tubulin, Sigma-Aldrich, catalogue number T5168
ErbB2, Abcam, clone CB11, catalogue number ab8054
EGFR, Abcam, clone EP38Y, catalogue number ab52894
PD-L1, Abcam, clone PDL1/2746, catalogue number ab238697
Hoechst, catalogue number 94403
Melittin, Monoclonal Antibody Facility at the Harry Perkins Institute of Medical Research, clone 3B9
Polyclonal goat anti-mouse IgG γ chain Antibody, Merck Millipore, AP503P
Goat anti-mouse Alexa Fluor 488-conjugated antibody, Thermo Fisher Scientific, catalogue number A11001
Goat anti-rabbit Alexa Fluor 594-conjugated antibody, Thermo Fisher Scientific, catalogue number A11012

Validation

All antibodies have been validated using positive and negative control conditions.

Eukaryotic cell lines

Policy information about cell lines

Cell line source(s)	American Type Culture Collection, Invitrogen (Australia), Asterand Bioscience, University of North Carolina in Chapel Hill and National Institutes of Health, Thermo Fisher Scientific (Victoria, Australia)
Authentication	Cell lines were acquired from reliable commercial and individual sources
Mycoplasma contamination	All the cell lines tested negative for mycoplasma contamination
Commonly misidentified lines (See ICLAC register)	*Name any commonly misidentified cell lines used in the study and provide a rationale for their use.*

Animals and other organisms

Policy information about studies involving animals; ARRIVE guidelines recommended for reporting animal research

Laboratory animals	Female A/J mice (anti-melittin antibody production), and Mus musculus, BALB/cJ, females, 4 weeks old (in vivo combinations)
Wild animals	The study did not involve wild animals
Field-collected samples	The study did not involve samples collected from the field
Ethics oversight	All animal protocols were approved by the Animal Ethics Committee of The Harry Perkins Institute of Medical Research, and the Animal Ethics Committee of The University of Western Australia

Note that full information on the approval of the study protocol must also be provided in the manuscript.

Flow Cytometry

Plots

Confirm that:

☒ The axis labels state the marker and fluorochrome used (e.g. CD4-FITC).

☒ The axis scales are clearly visible. Include numbers along axes only for bottom left plot of group (a 'group' is an analysis of identical markers).

☒ All plots are contour plots with outliers or pseudocolor plots.

☒ A numerical value for number of cells or percentage (with statistics) is provided.

Methodology

Sample preparation	Apoptosis and necrosis were assessed using the Annexin V-FITC Apoptosis Detection Kit I (BD Biosciences) according to the manufacturer’s protocol. SUM159 cells were plated in 6-well culture plates for 24 hours. Media was then discarded and replaced with media containing honeybee venom or melittin (IC50 concentrations) and cultured for 60 minutes. Cells were collected with trypsin and media, and centrifuged (1000 g, 5 mins, 24 ºC), washed with cold PBS, centrifuged (1000 g, 5 mins, 24 ºC) and re-suspended in 1X Binding Buffer. Cells were prepared to a concentration of 1 million cells/mL in 1X Binding Buffer. Samples were incubated with FITC and PI (5 µL of each) in the dark for 15 minutes. The presence of live, dead, apoptotic or necrotic cells was assessed using the BD Accuri C6 Flow Cytometer with BD Accuri C6 software.
Instrument	BD AccuriC6 (BD Biosciences, San Jose, USA)
Software	FlowJo™ Software (for Windows) Version 7.6.1. Ashland, USA
Cell population abundance	Not applied in this study
Gating strategy	Gating was defined according to the manufacturers instructions: Annexin V-FITC Apoptosis Detection Kit I (BD Biosciences)

☒ Tick this box to confirm that a figure exemplifying the gating strategy is provided in the Supplementary Information.
